# Ex vivo drug sensitivity screening predicts response to temozolomide in glioblastoma patients and identifies candidate biomarkers

**DOI:** 10.1038/s41416-023-02402-y

**Published:** 2023-08-24

**Authors:** Ioannis Ntafoulis, Anne Kleijn, Jie Ju, Kevin Jimenez-Cowell, Federica Fabro, Michelle Klein, Romain Tching Chi Yen, Rutger K. Balvers, Yunlei Li, Andrew P. Stubbs, Trisha V. Kers, Johan M. Kros, Sean E. Lawler, Laurens V. Beerepoot, Andreas Kremer, Ahmed Idbaih, Maite Verreault, Annette T. Byrne, Alice C. O’Farrell, Kate Connor, Archita Biswas, Manuela Salvucci, Jochen H. M. Prehn, Diether Lambrechts, Gonca Dilcan, Francesca Lodi, Ingrid Arijs, Martin J. van den Bent, Clemens M. F. Dirven, Sieger Leenstra, GLIOTRAIN consortium, Franck Bielle, Emie Quissac, Jane Cryan, Francesca Brett, Alan Beausang, Orna Bacon, Steve MacNally, Philip O’Halloran, James Clerkin, Martine L. M. Lamfers

**Affiliations:** 1 Department of Neurosurgery, Brain Tumor Center, Erasmus MC Cancer Institute, Erasmus MC, Rotterdam, Netherlands; 2 Department of Pathology & Clinical Bioinformatics, Erasmus MC, Rotterdam, Netherlands; 3 Information Technologies for Translational Medicine, Esch-Sur-Alzette, Luxembourg; 4 Luxembourg Centre for Systems Biomedicine, University of Luxembourg, Belvaux, Luxembourg; 5 Dept of Pathology and Laboratory Medicine, Legorreta Cancer Center, Brown University, Providence, RI USA; 6 Department of Internal Medicine, Elisabeth-Tweesteden Hospital, Tilburg, Netherlands; 7 DMU Neurosciences, Service de Neurologie 2-Mazarin, Sorbonne Université, Institut du Cerveau - Paris Brain Institute, Hôpital de la Pitié Salpêtrière, Paris, France; 8 Institut du Cerveau-Paris Brain Institute-ICM, Inserm, Sorbonne Université, CNRS, APHP, Hôpital de la Pitié Salpêtrière, Paris, France; 9 Department of Physiology and Medical Physics, Centre for Systems Medicine, Royal College of Surgeons in Ireland, Dublin, Ireland; 10 Department of Human Genetics, Laboratory for Translational Genetics, KU Leuven, and VIB Center for Cancer Biology, Leuven, Belgium; 11 Department of Neurology, Brain Tumor Center, Erasmus MC Cancer Institute, Erasmus MC, Rotterdam, Netherlands; 12 Department of Neuropathology, Beaumont Hospital, Dublin, Ireland; 13 National Centre of Neurosurgery, Beaumont Hospital, Dublin, Ireland

**Keywords:** Cancer models, CNS cancer, Predictive markers, Translational research

## Abstract

**Background:**

Patient-derived glioma stem-like cells (GSCs) have become the gold-standard in neuro-oncological research; however, it remains to be established whether loss of in situ microenvironment affects the clinically-predictive value of this model. We implemented a GSC monolayer system to investigate in situ*-*in vitro molecular correspondence and the relationship between in vitro and patient response to temozolomide (TMZ).

**Methods:**

DNA/RNA-sequencing was performed on 56 glioblastoma tissues and 19 derived GSC cultures. Sensitivity to TMZ was screened across 66 GSC cultures. Viability readouts were related to clinical parameters of corresponding patients and whole-transcriptome data.

**Results:**

Tumour DNA and RNA sequences revealed strong similarity to corresponding GSCs despite loss of neuronal and immune interactions. In vitro TMZ screening yielded three response categories which significantly correlated with patient survival, therewith providing more specific prediction than the binary MGMT marker. Transcriptome analysis identified 121 genes related to TMZ sensitivity of which 21were validated in external datasets.

**Conclusion:**

GSCs retain patient-unique hallmark gene expressions despite loss of their natural environment. Drug screening using GSCs predicted patient response to TMZ more specifically than MGMT status, while transcriptome analysis identified potential biomarkers for this response. GSC drug screening therefore provides a tool to improve drug development and precision medicine for glioblastoma.

## Background

Over the past 5 decades, great efforts have been put towards development of new anti-cancer therapies for the incurable brain tumour GBM. Promising preclinical results have been obtained for a plethora of new drugs, however, these results have not translated into improved outcomes in patients. One reason for this translational disconnect is that preclinical research in the past made use of clonal GBM cell line-based model systems that do not accurately reflect the molecular and phenotypic characteristics of these aggressive tumours [1]. The second cause of failure stems from the longstanding ambition in neuro-oncology to develop a one-size-fits-all treatment for GBM patients. However, multiple large cohort GBM characterization studies have demonstrated clinically-relevant intratumoral and interpatient variation with respect to driving mutations and targetable vulnerabilities [2, 3]. As such, clinically relevant screening models to address these variabilities may add valuable insights into trial stratification and responder identification to new treatments. Such models are expected to lead to the identification of effective therapies for subtypes of GBM and potentially even for individual patients. Focus has therefore shifted to the use of patient tumour tissue in preclinical research and culturing of patient-derived glioblastoma cells under serum-free conditions has now become the gold standard. A variety of in vitro models have been developed and these cultures are referred to as glioma stem(-like) cells (GSCs), brain-tumour initiating cells (BITCs), tumour neurospheres, or glioblastoma organoids. GSCs can be cultured in 2D on extracellular matrix or in 3D in non-adherent plates (neurospheres), whereas organoids are always grown in 3D. These models have been shown to preserve the core molecular and phenotypic signatures of GBM [1, 4–7] as well as its heterogeneity [4, 8, 9]. Additionally, when injected into mouse brains, GSCs or organoids give rise to invasively growing GBMs with characteristics of the parental tumour [5, 6, 10].

In keeping with these findings, we set up a drug-screening platform based on serum-free culture of low passage patient-derived GSCs in monolayers. Culture in 2D offers the advantage of rapid and straightforward assessment of drug sensitivity while preserving genomic stability and interpatient tumour subtype signatures [11]. In vitro cell culture, however, detaches GBM cells from their in situ microenvironment and it is becoming increasingly clear that interactions with local immune environment as well as neuronal networks and non-cellular components, contribute to the growth and survival of GBM [12–14]. It is therefore unknown to what extent a 2D in vitro drug screening assay on isolated GSCs can predict clinical efficacy of tumour-targeted agents and can ultimately support the identification of successful new treatment options. To assess this, we performed a retrospective analysis of patient and tumour-derived cell culture response to TMZ, the only chemotherapeutic agent currently integrated in the standard-of-care treatment [15, 16]. We correlated different in vitro readouts for TMZ response to the predictive biomarker MGMT (O6-methylguanine–DNA methyltransferase) promoter methylation status, as well as to clinical outcome after TMZ therapy. Furthermore, availability of both tumour and culture transcriptome data allowed identification of additional genes and pathways associated with this response.

The results of our retrospective study demonstrate the value of a simple and straightforward 2D assay on patient-derived GSCs for predicting clinical response to TMZ, despite the absence of the in situ microenvironment. Not only could responders and non-responders be identified but also an intermediate responding group, which offers a marked improvement to the binary classification by MGMT promoter methylation analysis. Moreover, our results suggest that this model may offer a tool in preclinical research and drug development to identify effective new tumour-targeted therapies for (subtypes of) GBM, as well as corresponding biomarkers of response. Taken together, our study provides support for further development of patient-derived GBM stem cell monolayer drug screening assays toward implementation in preclinical drug development programs as well as precision medicine approaches.

## Materials and methods

### Patient selection and outcome

GSC cultures were obtained from our biobank containing tissue and derived cell cultures from primary brain tumour samples from the Erasmus Medical Centre, Rotterdam and Elisabeth Tweesteden Hospital, Tilburg, both in the Netherlands. The use of patient tissue for this study was approved by the local ethics committees of these hospitals and all patients signed informed consent forms according to the guidelines of the Institutional Review Boards of the respective hospitals. Tumour classification was performed by the local neuropathologist according to guidelines of the WHO 2007 and 2016 classification of primary brain tumours. The biobank was searched for samples obtained between September 2009 and May 2019 matching the following criteria: primary glioblastoma (no recurrent), IDH wild type, fresh frozen tumour material available, successful GSC culture below passage 10 available, patient received standard treatment (radiotherapy with concomitant and adjuvant temozolomide [15]), and availability of complete follow-up data. Of the 421 glioblastoma IDH wildtype tissue samples received in the lab, 66 GSC cultures were ultimately available derived from patients treated with Stupp regimen (Supplementary Fig. 1).

As primary endpoints for outcome, we used progression-free survival (PFS) and overall survival (OS). PFS was calculated from the day of operation to the first evidence of radiological and/or clinical progression, as concluded in radiology reports of follow-up MRI scans. OS was calculated from the day of operation until death.

### Tumour processing and cell culture

Fresh glioma tissue samples obtained directly from the operating room were processed according to protocols previously described to obtain monolayer GSC cultures under serum-free conditions [17, 18]. Details are available in the supplementary methods. Samples not matching parental tumours or infected with mycoplasma were removed from further analysis (Supplementary Fig. 1). GSC cultures below passage 10 were used, as heterogeneity of patient-derived GSC cultures is reported to decrease at higher passage numbers [19].

### Methylation-specific polymerase chain reaction PCR for MGMT

DNA was extracted from snap frozen primary tumour material and the derived GSC cultures using the QIAamp DNA mini kit (Qiagen) according to manufacturer’s instructions. Isolated DNA was modified with sodium bisulphite using the EZ DNA Methylation Gold^TM^ kit (Zymo Research, Baseclear) and used as a template for methylation specific (MS) PCR. Primers specific for methylated and unmethylated MGMT promoter DNA and PCR conditions were used as described previously [19–21]. This procedure is used on a daily basis for patient diagnostics.

### In vitro temozolomide sensitivity testing

Each of the 66 GSC cultures was screened for temozolomide (Sigma-Aldrich) sensitivity according to the following protocol (Supplemental Fig. 2). Cells were seeded onto 96-well plates coated with extracellular matrix (BD Bioscience) at 1000 cells/well. A stock solution of Temozolomide was diluted in serum-free culture medium to obtain a starting concentration of 400 μM in 0.4% DMSO, which was further diluted in twofold steps. For controls, the same 2-fold dilution steps were applied starting with 0.4% DMSO. Serial dilutions of TMZ and DMSO controls were added after 24 h and cell viability was assessed by CellTiter GLO 2.0 after six days (Promega). For details see supplementary methods. For each of the 66 GSC cultures, one biological and three technical replicates were included for the TMZ and DMSO dose-range. Using the same set up, reproducibility of the assay by different operators was confirmed in 40 cell cultures, in which TMZ sensitivity was determined by two or three different operators over multiple years, and which revealed acceptable to excellent coefficient of variation in 90% of the samples. (Supplementary Table 2).

### DNA sequencing

DNA extracted from 56 primary GBM samples and of 19 derived cell cultures was isolated using DNeasy Blood and Tissue kit (Qiagen) as described previously [22]. The DNA libraries were prepared using the KAPA library prep kit for Illumina (KAPA HTP Library Preparation Kit for low coverage whole genome sequencing (LC-WGS)) and sequenced on an Illumina HiSeq4000 using a flow-cell generating 1 × 51 bp single reads, aiming for 5,000,000 unique reads per sample. Raw reads were mapped to the human reference genome (hg19) using Burrows-Wheeler Aligner version 0.7.12 [23], duplicate reads were removed using Picard tools (version 1.43) (http://broadinstitute.github.io/picard) and copy-numbers profiles were generated by binning reads in windows of 50 kb using QDNAseq [24]. The resulting log R-values were segmented with the ASCAT algorithm (v2.0.7) [25].

### RNA sequencing

RNA of 56 primary GBM samples and of 19 derived cell cultures was isolated using Trizol (ThermoFisher Scientific) as described in supplementary methods. RNA libraries were prepared from 2 ug total RNA, using KAPA stranded mRNA library prep and sequenced single-end 50 bp on an Illumina HiSeq 4000. Before mapping, the optical duplicates and adaptors were removed with clumpify and fastx-toolkit, respectively. Next the reads were mapped to the human reference genome GRCh38 with STAR2.6 and gene-expression matrices were generated with HTSeq. The cell line RNA sequencing data were preprocessed using R package “EdgeR” [26]. Low expressed genes were first filtered out using the function “filterByExpress” after which the raw count matrix was normalized using TMM method in log2 scale (“CalcNormFactors” function).

### Correlation analysis of DNA and RNA sequences between cell cultures and matched tissues

Matched tissue and cell culture correlation analyses were computed using the “cor.test” function in R with Pearson method. The correlation of the copy number variation was performed on normalized logR values associated to the same chromosome regions, corresponding to 81.4% of the sequenced regions. The correlation of gene expression was performed on the normalized gene expression values. All *p* values were corrected for multiple comparison using the False Discovery Rate (FDR).

Gene Set Enrichment Analysis (GSEA) and over-representation analysis utilizing Enrichr API was performed in python with the use of GSEApy [27]. In short, the previously normalized transcriptomic data of matching tissue and cell culture samples, containing only genes shared across the two biological groups, underwent GSEA utilizing the Enrichr libraries MSigDB_Hallmark_2020, GO_Biological_Processes_2021, GO_Molecular_Function_2021, and GO_Cellular_Component_2021 [28–30]. A combination of the nominal (nom) *p* value, false discovery rate (FDR) q-value, and family-wise error rate (FWER) *p* value were used to determine the significance of a gene set normalized enrichment score (NES). Seaborn cluster mapping capabilities were used to elucidate and visualize further structure in GSEA data [31]. To further understand the relationship between cell culture and tissue data, violin plots were generated via Seaborn to compare the expression of hallmark GBM genes across model systems. Wilcoxon signed-rank statistical testing and Spearman’s correlation analysis was done via SciPy [32]. In addition, GSEApy’s Enrichr based over-representation analysis was used to perform GO enrichment analysis on genes uniquely present in either tissue or cell samples utilizing the GO libraries available on Enrichr. Further details are available in the Supplementary methods.

### Correlation analysis of the TMZ response with RNA-seq data of cell cultures and tumour tissues

Differential expression analysis was conducted to compare the expressed genes in cell culture and tissue RNA-seq data. The cell culture data (*n* = 19) was categorized based on % viability at 100 uM TMZ, while the patient tissue samples (*n* = 56) were categorized into three groups: responders, intermediates, and non-responders based on overall survival. Lowly expressed genes were filtered out before identifying DE genes using the DESeq2 package [33] in R. Spearman correlation analysis was performed on the significant DE genes of cell culture data to determine genes correlated with TMZ response, using the R function “cor.test” [34]. Cox proportional hazards analysis was conducted using the with R function “survival” [35, 36], on the significant DE genes of tissue data to identify genes with an effect on survival or TMZ response in patients. To validate the overlapping genes between cell culture and tissue data sets, a Cox proportional hazards analysis was conducted on two additional cohorts (the GLIOTRAIN dataset and TCGA dataset). For details see supplementary methods. The pathway analysis was carried out in the Gene Set Enrichment Analysis (GSEA) program [37], using the Hallmark gene sets from Molecular Signatures Database (MSigDB). The cut-off values for significantly correlated pathways were set at FDR *q*-values < 0.15. For details see supplementary methods.

### Statistical analysis

GraphPad Prism software version 9 (GraphPad Software) was used for defining IC_50_, percentage viability and Area Under the Curve (AUC) values and related statistical analysis. Nonlinear regression (curve-fit) and the equation [inhibitor] vs normalized response with variable slope model was applied to calculate the IC_50_ values. The reported IC_50_ is the concentration of TMZ that gives a response half way between the highest and lowest value of each curve. In some cases this value may deviate from Y = 50. Survival curves were generated using GraphPad Prism and survival analyses were performed using SPSS (IBM Statistics 28.0.1.0). Student’s *t* test was used to correlate in vitro sensitivity of TMZ with MGMT status. *P* values below 0.05 were considered significant for these analyses.

## Results

### Molecular comparison of GBM tissues and derived GSC cultures

**Fig. 1:**
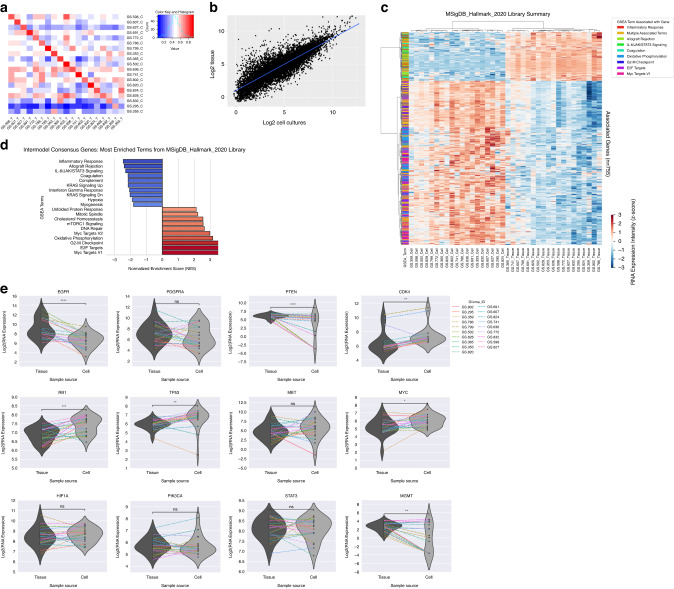
Correlation of molecular features of parental tissues and derived cell cultures. **a** Correlation analysis of DNA sequences between the tumours and their corresponding cell cultures based on the normalized logR ratios (*n* = 19) (mean Pearson’s r = 0.77). The values represent the correlation coefficients. Samples names with “C” indicate cell cultures, while “T” indicate tissues. **b** Correlation analysis of the log2 averaged gene expression between tumours and corresponding cell cultures (*n* = 19) (Pearson’s r = 0.76, *p* < 0.0001). The blue line represents the trendline of the linear regression. **c** Gene Set Enrichment Analysis (GSEA) clustermap illustrating differential expression of bulk RNAseq data between corresponding tissue (*n* = 19) and cell cultures (*n* = 19) in reference to the MSigDB_Hallmark_2020 library extracted from Enrichr. Genes from the top 4 terms containing the most negatively and positively normalized enrichment scores (NES) are represented here (*n* = 755). **d** GSEA illustrates the top 20 terms with the most positively (*n* = 10) and negatively (*n* = 10) NES. Blue indicates terms with negative enrichment scores and red positive enrichment scores. Intensity of the colour is indicative of relative significance (all terms present are NOM p-val, FDR q-val, FWER *p* val significant). **e** Violin plots illustrating mean expression of hallmark genes between tissue and cell cultures. Each line indicates an individual paired sample. *P* values < 0.05 were considered significant.

To establish whether in vitro culture systems retain the genetic programs of matched tumours, total DNA and RNA was isolated and sequenced from a panel of tumours and derived cell cultures (*n* = 19). Correlation analysis was performed to assess similarity between the paired samples. Results revealed that DNA sequences remained largely conserved between the tumours and their corresponding cell cultures as shown in Fig. 1a and as individual plots of paired samples in Supplementary Fig. 3 (Pearson’s mean r = 0.77, median r = 0.77, range 0.48–0.92, Supplementary Table 3A).

Transcriptome comparison also revealed a strong correlation between cell cultures and patients’ tissues. Mean overall Pearson’s correlation coefficient between all tissues and all cultures was r = 0.76 (*p* < 0.0001) (Fig. 1b). The individual correlations coefficients of tissue-culture pairs also revealed high correlations coefficients ranging from r = 0.64–0.86 (median 0.77) (Supplementary Table 3B and Supplementary Fig. 4). As expected, distinct changes were also noted between parental tissue and derived cultures. Unsupervised clustering of the 18,058 genes commonly expressed between the parental tumours and derived cell cultures (consensus genes) demonstrates that there is a clear delineation between cell cultures and tissues. Two gene clusters show an inverse relationship and a third demonstrating no clear delineation (Supplementary Fig. 5). GSEA of the two data sets, utilizing the MSigDB Hallmark 2020 as a reference, found cell culture samples to possess higher expression of genes relating to cell cycle replication, including G2M checkpoint, E2F and MYC targets [38]. Conversely, tissue samples displayed an increased expression in genes relating to immune processes including inflammatory responses and IL-6/JAK/STAT pathway (Fig. 1c, d). Further GSEA analysis across 3 additional reference libraries (GO series) reinforces this data. Tissues contain increased expression of cytokine, lysosome and MHC complex related processes. In contrast, cell cultures contain increased expression of nuclear, chromosome, RNA, and DNA related genes (Supplementary Fig. 6A–C). These trends are further reinforced when analysing the genes unique to tissue samples, where an overrepresentation of genes implicated in neuronal intercellular communication were identified, including serotonin, calcium and chloride channels, as well as neuropeptide activity. Furthermore, processes related to cytokine and chemokine activity as well as regulation of immune response were identified (Supplementary Fig. 6D).

Whether these changes affect the expression patterns of hallmark genes in GBM between tissues and derived cultures, was assessed by comparing expression levels of the hallmark genes EGFR, PDGFRA, PTEN, CDK4, RB1, TP53, MET, MYC, HIF1A, PIK3CA, STAT3 as well as MGMT (Fig. 1e). A complete list of hallmark genes is provided in Supplementary Table 4. For some genes, including EGFR, significant up or downregulation of the mean expression was noted between tissues and derived cell cultures. However, the interpatient expression profiles were generally well-maintained between the sample sets, as noted by the limited number of crossings of connector lines and the fact that there were no significant negative Spearman correlation values. However, results from genes based on CDK6, FGR1 and BCL2 may need to be interpreted with caution (Supplementary Table 4).

Taken together, our data implies that tumour samples undergo a transcriptomic shift from intercellular communication in situ to increased cell cycle and growth activity in vitro. Despite these changes, hallmark gene expression profiles are maintained in a patient-specific manner.

### Sample selection and analysis of representativeness of the cohort

To assess whether these changes in microenvironment affect the potential of ex vivo glioblastoma cultures to predict response to treatment, we retrieved tumour samples and derived GSC cultures from our glioma biobank for a retrospective study to correlate in vitro response to TMZ to clinical response. We identified 66 samples fulfilling the inclusion criteria of this study (Supplementary Fig. 1). The median age of the 66 patients in our cohort was 61.5 (35.4–81.0) years (Supplementary Table 1). In all patients, radiotherapy with concomitant TMZ treatment was initiated between 4–6 weeks after surgery. Eleven of the 66 patients received a short-course chemoradiation schedule by physicians’ choice (mainly because of age >60 and/or low Karnofsky performance score) and with the exception of one patient no adjuvant cycles of TMZ. The remaining 55 patients received a median of 5 adjuvant TMZ cycles. Median PFS and OS of the 66 patients was 6.65 (1–36.4) months and 13.69 (2.8–37.4) months, respectively (Supplementary Table 5).

The MGMT promoter methylation status was determined for all tumour and cell culture samples included in the study. The distribution of this prognostic and predictive tumour biomarker within our cohort was 47% methylated and 53% unmethylated. Median PFS and OS of the MGMT methylated group was 8.55 and 17.19 months, respectively, versus 5.92 and 12.4 months in the unmethylated group (Supplementary Table 5). These results are comparable to the results of the Stupp trial [16], indicating that our cohort is representative for the GBM population.

### MGMT status is preserved in the majority of primary GSC cultures and predicts in vitro response to TMZ

**Fig. 2:**
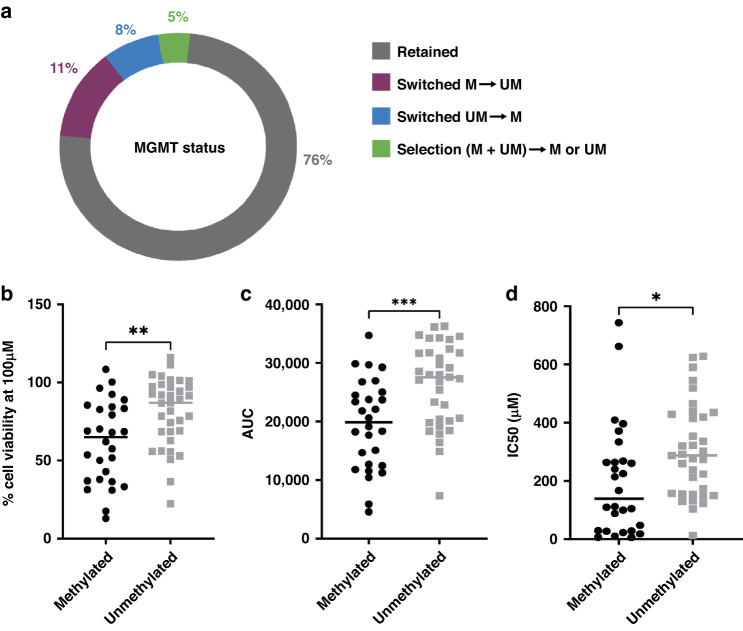
MGMT status of primary tumours is generally maintained in derived GBM cultures and related to in vitro response to TMZ. **a** Percentage of GSC cultures (*n* = 64) with maintained MGMT promoter methylation status (Retained), switched from methylated to unmethylated or vice versa (Switched) or selection from mixed to single signal (Selection). **b** In vitro response of TMZ (expressed as % cell viability at 100 uM, y axis) compared to the MGMT status of GSC cultures. **c** In vitro response of TMZ (expressed as AUC, y axis) compared to the MGMT status of GSC cultures. **d** In vitro response of TMZ (expressed as IC_50_, y axis) compared to the MGMT status of GSC cultures. Asterisks indicate significantly different means at the indicated read-out (unpaired *t* test, ***p = 0.0006, **p = 0.002 and *p = 0.02). *P* values < 0.05 were considered significant.

The MGMT status was available for 64 of the 66 GSC cultures and compared to the parental tumours. This revealed that in 49 out of 64 cases (76%) the MGMT status was preserved. In 5 cases MGMT was unmethylated in the parental tumour, whereas it was found methylated in the derived cell culture (UM→M, 8%). In 7 cases, MGMT promoter methylation in the tumour was lost in culture (M→UM, 11%). A small percentage of samples revealed a selection from a mixed signal in the tumour to a homogeneous signal in culture (5%) (Fig. 2a).

Dose-response analysis was performed upon screening of temozolomide on the panel of 66 primary GBM cultures (Supplementary Fig. 7). Based on the results, three readouts for TMZ efficacy were determined: IC_50_ values, area under the curve (AUC) and percentage cell viability at 100 μM. This concentration is close to the maximum reported cerebrospinal fluid (CSF) concentration of 75 μM for TMZ and was previously found to differentiate between sensitive versus resistant GSC cultures to TMZ in our drug testing model [39, 40].

As expected, MGMT methylated GSC cultures displayed higher sensitivity to TMZ than unmethylated cultures in all three readouts, with AUC revealing the largest difference (Fig. 2b–d). Contrary to other anti-cancer agents tested in this platform [18], TMZ treatment can yield a steep dose response curve within the effective range (25–300 μM) (Supplementary Fig. 7), generating larger variability in calculated IC_50_ values between replicates. Moreover, for highly resistant cultures which retain >50% viability at the highest TMZ dose (16 of 66 samples), the IC_50_ values are based on extrapolation and are therefore less reliable. As a result, using the percentage cell viability and AUC values as readouts, yields more robust results which also show stronger correlation to MGMT status.

### In vitro response to TMZ is predictive for clinical response of corresponding patients

**Fig. 3:**
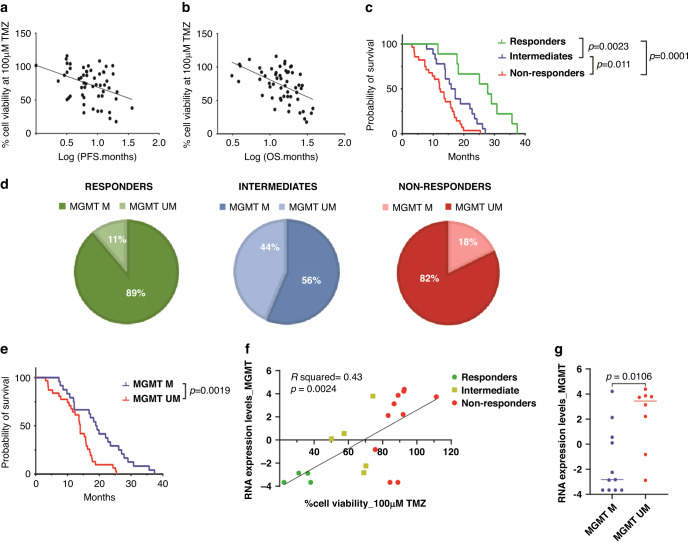
In vitro response to TMZ predicts clinical response of corresponding patients. Correlation analysis of the percentage cell viability at 100 uM TMZ (y-axis, percentage of DMSO control) of GSC cultures (*n* = 55) with (**a**) progression free survival (x-axis, PFS in months) or (**b**) the overall survival (x-axis, OS in months) of the corresponding patients (Spearman’s rank correlation). **c** Survival graph of patient cohort (*n* = 55), comparing overall survival (x axis, OS in months) to TMZ in vitro response as classified by responders (*n* = 9, green), intermediates (*n* = 18, blue) and non-responders (*n* = 28, red), Responders vs intermediate (log-rank *p* = 0.0023), intermediate vs non-responders (log-rank *p* = 0.0082) and responders vs non-responders (log-rank *p* < 0.0001). **d** Distribution of MGMT status of GSC cultures of the three response groups (responders, intermediates and non-responders). **e** Survival graph of patient cohort (*n* = 55) comparing survival (x-axis, OS in months) to cell culture MGMT status, unmethylated (red) vs methylated (blue) (log-rank *p* = 0.0019). **f** Correlation of MGMT RNA expression level to % cell viability at 100 μM of TMZ (R^2^ = 0.43, *p* = 0.0024). **g** Comparison of MGMT RNA expression level to the MGMT promoter status (M and UM) (*p* = 0.0106). *P* values < 0.05 were considered significant.

Two types of analyses were performed to assess whether in vitro response to TMZ correlates with clinical outcome of patients receiving Stupp protocol. Patients who received short-course radiotherapy plus TMZ (*n* = 11) were excluded from analysis to increase the uniformity of the cohort with regard to other prognostic factors such as age or Karnofsky Performance Score (KPS). For the remaining 55 patients, we performed Spearman correlation analysis and Cox regression between the in vitro response data (IC_50_, AUC, and % viability at 100 μM values) and their clinical outcome (PFS and OS). For the Spearman correlation analysis, % viability at 100 μM gave the strongest correlation with both PFS (r = −0.38, *p* = 0.014) and OS (r = −0.495, *p* = 0.0002) (Fig. 3a, b).

AUC also correlated significantly with PFS (r = −0.29, *p* = 0.03) and OS (r = −0.399, *p* = 0.0026) whereas IC_50_ values revealed non-significant correlation with PFS (r = −0.206, *p* = 0.132) but significant correlation with OS (r = −0.35, *p* = 0.009) (Supplementary Fig. 8). Cox regression analysis also revealed statistically significant correlation of % viability at 100 μM with PFS (*p* = 0.012) and OS (*p* < 0.00005) (Supplementary Table 6).

The distribution of the in vitro results revealed a gradient in TMZ response. We therefore visualized the correlation of in vitro response (%viability) vs. OS by Kaplan-Meier analysis using 3 groups to emphasize that the response is non-binary. Dividing the group by quartiles of % viability into responders (<50%), intermediate responders (50–75%) and non-responders (>75%) at 100 μM TMZ, revealed significantly different survival curves for the three groups (median survival of 27.8, 15.9, and 12.3) months for responders (R), intermediates (I) and non-responders (NR), respectively (log-rank R vs NR *p* = 0.0001, R vs I *p* = 0.0023, and I vs NR *p* = 0.011) (Fig. 3c). This segregation is again confirmed by the Cox regression analysis, where 3-group segregation was used as the covariate (*p* < 0.00005). The outcome of responders and non-responders generally aligned with the cell culture MGMT status, however, for 33% of patients (the intermediates) cell culture MGMT status did not predict response to TMZ (Fig. 3d). The patients categorized in these response groups did not significantly differ in terms of age or KPS scores, nor in number of adjuvant TMZ cycles received or extent of tumour resection (Supplementary Fig. 9). Segregation of the group by quartiles of AUC values (<50%, 50–75%, >75%) or IC_50_ values (<100, 100–300, >300 uM TMZ) did not significantly differentiate non-responders from intermediates but did discriminate responders from these groups (Supplementary Fig. 10, Supplementary Table 6). Segregation of the cohort by tumour MGMT status revealed a less significant and less specific overall survival prediction (*p* = 0.001) (Fig. 3e). For 19 samples, we also assessed whether MGMT gene expression levels are better correlated to TMZ response than MGMT promoter methylation status. This revealed slightly more consistency between the MGMT expression levels and the response groups (*p* = 0.002) than between the MGMT levels and promoter methylation status (*p* = 0.011). Thus, compared to the established biomarker MGMT methylation status, which postulates a bimodal response to TMZ, our assay provides a means to separate patients more specifically into three distinct response categories.

**Fig. 4:**
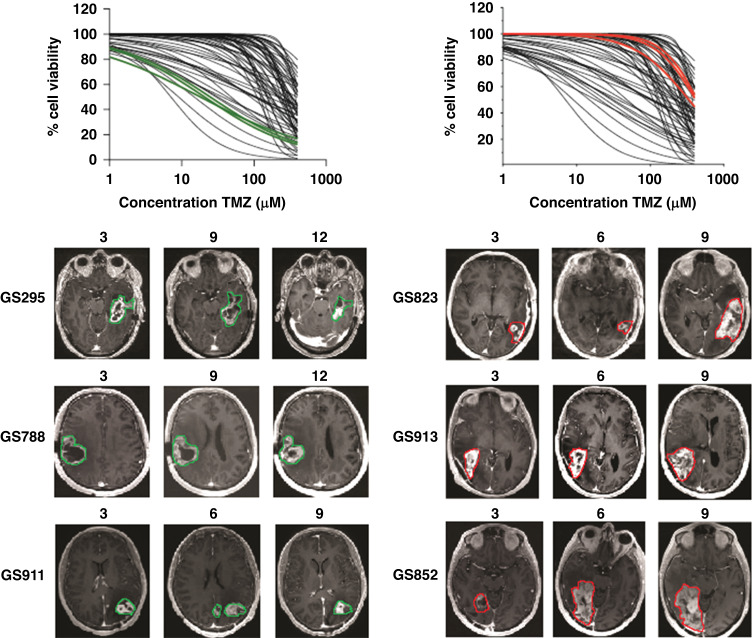
In vitro sensitivity of TMZ predicts treatment response in patients who received TMZ/RTx. Representative examples of consecutive T1 weighted post gadolinium MRI images of 6 patients, which were defined as 3 predicted responders (cell viability below 50%, green curves, left graph) and 3 non-responders (cell viability above 75%, red curves, right graph), showing stable disease in the responder patients (left) and progressive disease in the non-responder patients (right). Tumour area was delineated in all MRI scans, responders (green) and non-responders (red).

To further substantiate these results, MRI images at follow-up time points post TMZ/RTx treatment were collected from 3 predicted responders (viability below 50%) and 3 predicted non-responders (viability above 75%). As shown in Fig. 4, clear tumour progression is seen between 3 and 9 months after surgery (during TMZ/RTx treatment) in the non-responder patients. In the responder patients, the residual tumour after surgery remains stable during TMZ/RTx treatment (GS295, GS911) (Fig. 4). Altogether, our results indicate that in vitro TMZ response data, in particular % cell viability at 100 µM, is predictive of treatment response to TMZ/RTx in patients.

### Transcriptome analysis of cell cultures identifies markers in relation to TMZ response

**Fig. 5:**
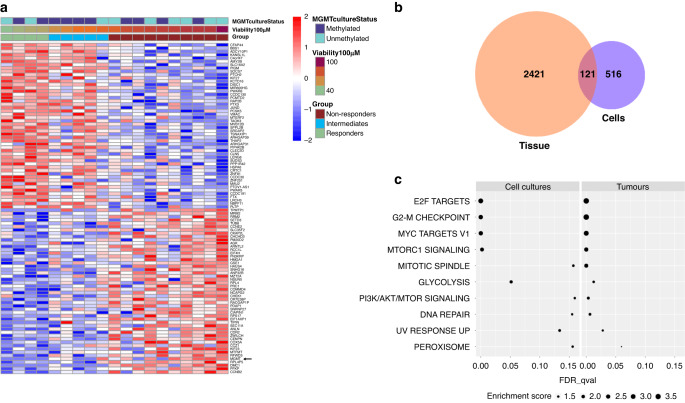
Transcriptome correlations between in vitro and patient response to TMZ. **a** Heatmap of the top 100 signature genes correlated with TMZ response in terms of the % viability at 100 uM for the 19 GBM cell cultures (*p* value < 0.05). Each row of the heatmap represents one significant gene, while each column represents one sample. The genes are ordered by the coefficients of the Spearman correlation analysis. The samples are ordered by the values of the TMZ response outcomes. **b** Venn diagram describes the significant correlated genes to TMZ response in both tissue and cell culture datasets as well as the overlapping genes. **c** Overlapping pathways in cell cultures (left) and the tumours (right) (False discovery rate <0.25). The FDR *p* values on the x-axis show the significance of the pathways in cell cultures/tumours, while the size of the circles represents the enrichments score of each pathway in each category. The pathways are ordered by the averaged enrichment scores of the cell cultures and tumours.

Drug response data was compared to transcriptome data obtained for 19 GSC cultures to identify genes related to in vitro response to TMZ. This analysis identified 637 differentially expressed genes significantly correlated to TMZ response, including MGMT (Spearman’s r = 0.71, *p* = 0.0006) (Supplementary file 1). Figure 5a depicts a heatmap in which the top 100 signature genes, 50 for resistance and 50 for sensitivity, are plotted ranked by the coefficients of the correlation analysis. MGMT is marked by an arrow and the response groups are marked at the top in green (R), blue (I) and red (NR). Notably, the responders clearly show the most differential gene signature compared to the other samples. Distribution of the established GBM transcriptomic subtypes (classical, mesenchymal, proneural) within TMZ response groups revealed a slightly higher number of mesenchymal-like GBM cases in the non-responders (6 of 10) compared to responders (2 of 4). The only 3 proneural GBM cases were grouped in the non-responders (Supplementary Fig. 11).

### Validation of cell culture response signatures with tissue transcriptomic data

Transcriptome data of tumour tissue of the GLIOTRAIN Erasmus MC samples (*n* = 56), of which *n* = 19 corresponding to the cell cultures, was correlated to the patients’ overall survival. Of note, all patients in the GLIOTRAIN cohort received TMZ + radiation therapy. This analysis revealed 2542 differentially-expressed genes which significantly correlated to survival, including MGMT (Hazard ratio = 1.85, *p* < 0.0001) (Supplementary Fig. 12, Supplementary file 1). Interestingly, MGMT promoter methylation status did not correlate with OS (*p* = 0.16). Of the 2542 genes, 121 genes overlapped with the genes correlated to TMZ response in the in vitro data set (Fig. 5b, Supplementary file 1).

Pathway analysis performed on the 2542 differentially expressed genes from the tumours and the 637 genes from GSC cultures identified 10 significant overlapping pathways, many of which have previously been related to TMZ resistance including; (i) DNA repair, (ii) G2M check point, (iii) E2F targets (iv) PI3K/Akt/mTOR signalling and (v) myc targets (Fig. 5c, Supplementary file 1).

Validation of the identified genes related to TMZ response was performed on two additional data sets. We compared our gene list to the remaining GLIOTRAIN (non-Erasmus) samples not included in the initial analysis (*n* = 70) as well as to the publicly-available TCGA data considering only GBM patients who received TMZ treatment (*n* = 89). Within the GLIOTRAIN data set, 16 of 121 genes were significantly correlated to survival, including MGMT (HR1.21, *p* < 0.024) (Table 1A). Likewise, the TCGA data provided 6 significantly correlated genes. One overlapping gene between both cohorts was identified, the transcriptional repressor Zinc Finger protein 540 (ZNF540) (HR 0.66, *p* = 0.025) (Table 1B). Interestingly, in the GLIOTRAIN cohort 13 of 16 identified genes were related to improved survival (HR < 1) versus only 1 of 6 in TCGA. This may be related to the fact that GLIOTRAIN samples were selected to contain a relatively high percentage of long-term (>36 months) survivors (30%) compared to TCGA (5.6%), whereas TCGA had more short-term (<9 months) survivors (42.7%) compared to GLIOTRAIN (7%). All together, these results demonstrate that in vitro sensitivity testing on molecularly characterized cell cultures can help identify signatures related to drug response, which in the case of TMZ could be validated on external data sets.

**Table 1:** Gene lists external validation.

A. GLIOTRAIN data set				
Gene	Gene Name	Hazard ratio	*P*. value	P. adj
RPS2	Ribosomal Protein S2	1.91	0.0167	0.144
APRT	Adenine phosphoribosyl transferase	1.63	0.0391	0.263
MGMT	O-6-Methylguanine-DNA Methyltransferase	1.21	0.0248	0.179
ZNF471	Zinc Finger Protein 471	0.69	0.0149	0.138
ZNF540	Zinc Finger Protein 540	0.67	0.0197	0.159
ZMAT1	Zinc Finger Matrin-Type 1	0.64	0.0081	0.089
ZNF585B	Zinc Finger Protein 585B	0.60	0.0013	0.019
ZNF493	Zinc Finger Protein 493	0.60	0.0060	0.073
AMY2B	Amylase Alpha 2B	0.58	0.0108	0.109
CLEC2D	C-Type Lectin Domain Family 2 Member D/LLT1	0.56	0.0003	0.005
ZNF224	Zinc Finger Protein 224	0.54	0.0001	0.003
ZNF547	Zinc Finger Protein 547	0.52	0.0002	0.003
SWT1	SWT1 RNA Endoribonuclease Homologue	0.48	0.0001	0.003
RNPC3	RNA Binding Region (RNP1. RRM) Containing 3	0.43	0.0016	0.021
FTX	Long noncoding RNA FTX	0.38	0.0001	0.003
PSMA3-AS1	PSMA3 Antisense RNA 1	0.28	0.0001	0.003

B. TCGA data set				
Gene	Gene Name	Hazard ratio	*P*. value	P. adj
ARPC1A	Actin Related Protein 2/3 Complex Subunit 1A	2.57	0.01	0.68
NTAN1	N-Terminal Asparagine Amidase	2.01	0.04	0.71
RPP25	Ribonuclease P and MRP Subunit P25	1.44	0.03	0.68
ANLN	Anillin. Actin Binding Protein	1.39	0.03	0.68
SYNJ2	Synaptojanin 2	1.31	0.03	0.68
ZNF540	Zinc Finger Protein 540	0.67	0.03	0.68

External validation of genes significantly correlated to survival of TMZ/RTx-treated GBM patients

A) Table showing the *n* = 16 candidate genes related to TMZ response which correlated to GBM patient survival in the GLIOTRAIN data set.

B) Table showing the *n* = 6 candidate genes related to TMZ response which correlated to GBM patient survival in the TCGA data set. *P* values < 0.05 were considered significant.

## Discussion

In this retrospective study we assessed whether loss of interactions between GBM cells and their tumour microenvironment affect the predictive value of a 2D functional screen testing a clinically-relevant pharmaceutical intervention. To do this, we charted the transcriptomic differences between GSC cultures and corresponding parental tumours, and we assessed the feasibility of the GSC monolayer screen to predict clinical efficacy of TMZ for GBM patients.

Correlation analysis of DNA and RNA sequences from low-passage GBM cultures with parental tissues revealed in general a strong correlation between paired samples, including expression profiles for hallmark genes of GBM. However, transcriptomic comparison also revealed marked differences, with pathways related to neuronal intercellular communication and immune cell interactions being exclusive for tissues, and pathways related to cell cycle processes being upregulated in cell cultures. The origin of this transcriptomic shift can be attributed to the selection of cells from the in situ tumour environment that have an intrinsic survival advantage in vitro, as well as the adaptation of these cells to 2D culture condition. With the growing body of evidence that tumour micro-environmental interactions play an important role in GBM growth and development in situ [12–14], the question is raised whether drug testing on patient-derived isolated tumour cells can predict patient response to a tumour-targeted treatment.

To address this, we implemented a simple and straightforward 2D drug screening approach, which allowed accurate and reproducible drug efficacy assessment [40]. This offers an advantage to 3D model systems where precise cell numbers and live-dead ratios can be difficult to determine. Screening of 55 low-passage GSC monolayer cultures for TMZ sensitivity revealed a robust correlation between in vitro effects and survival of patients receiving treatment with TMZ (Figs. 3, 4). Significant correlations to PFS and OS were found using either AUC or percentage cell viability after treatment with 100 μM TMZ, a dose selected based on therapeutic ranges found in cerebral spinal fluid of TMZ treated patients [39]. Our assay is the first functional screen to reveal 3 categories of TMZ response which significantly correlate with patient survival. Known confounders, unrelated to the 2D-monolayer model, such as age, physical condition (KPS) or extent of tumour resection were not significantly different between the response groups. This classification into three groups offers a marked improvement to the established dichotomous biomarker for TMZ response, the MGMT promoter methylation status.

As expected, TMZ was more effective in MGMT methylated than unmethylated cell cultures (Fig. 2b–d), confirming previous studies [5, 41]. However, considerable inter-tumoral variability in TMZ sensitivity within both groups was observed. This finding is corroborated by the discovery of an intermediate response group in patients which contains a near-equal distribution of methylated and unmethylated samples (Fig. 3d). Indeed, gene expression levels of MGMT also revealed a gradient, with intermediate expression related to intermediate response to TMZ (Fig. 3f), indicating that MGMT is not expressed in a binary manner. MGMT gene expression levels also correlated better to OS than the promoter methylation status, as clearly noted in the GLIOTRAIN cohort (Supplementary Fig. 12). Resistance to TMZ in the MGMT methylated group can also be explained by other factors related to TMZ response, such as deficiency in functional DNA mismatch-repair (MMR) [42]. Additionally, *HOX* signature, *EGFR* expression, as well as base excision repair enzymes have been linked to TMZ resistance [43, 44]. Altogether, these factors indicate that MGMT promoter methylation status alone is not sufficient for predicting response to TMZ and that a direct ex vivo functional test may provide a more accurate prediction. In this context, functional screening can also aid in selecting patients who are not expected to benefit from TMZ therapy. These patients could be spared the burden of unnecessary toxicity and offered alternative treatments or enrolment in clinical trials in the up-front setting, an increasingly applied strategy in GBM clinical research [45].

The use of patient-derived ex vivo tumour models for drug testing may thus aid in treatment selection. Such approaches are being undertaken for various types of cancer and the generated data confirm the predictive power of patient-derived in vitro assays, including GBM [46–49]. Howard et al. described a technology combining in vitro TMZ sensitivity data from separately cultured bulk tumour and bioreactor-cultured stem cells which allowed prediction of patient response to TMZ [50]. More recently, Stockslager et al. described an interesting approach to predict patient TMZ response based on GSC single-cell mass [51]. Both previous studies confirm the predictive power of ex vivo drug testing assays for GBM, however, the applied techniques and analysis tools may not be easily translated to routine laboratory and clinical use. In this respect, our simple monolayer culture with ATP-based viability read-out as detailed in this paper offers an important advantage.

The clinical relevance of TMZ testing in our GSC monolayer assay, also prompted us to investigate whether molecular markers related to TMZ sensitivity could also be identified using this system. The pathway analysis identified 10 pathways present in both cell culture and tumour data relating to TMZ response. Interestingly, many of these pathways have previously been reported to play a role in TMZ resistance mechanisms. DNA repair, G2/M checkpoint and E2F targets are closely related cellular processes and important players in TMZ resistance by regulating the cell cycle and allowing cells to repair genomic damage [52]. Downstream effects of the transcription factor Myc have also been reported to drive TMZ resistance [53], including the identified Myc targets CDK2, PHB2 and PRDX3 [54–56]. Of the 121 identified genes, 16 could be validated in the GLIOTRAIN data set, including MGMT, and 6 in the TCGA data set. One overlapping gene was identified, ZNF540, which is a member of the ZNF protein family reported to bind to major vault protein to inhibit the ERK signalling pathway [57]. It was previously also identified in a study into multi-gene signatures to predict prognosis and treatment response in GBM [58]. Together, these findings support our hypothesis that functional screening in patient-derived GBM models can offer a tool to identify candidate novel therapies and corresponding signatures of response. Larger scale studies, powered for biomarker discovery, are required to validate our biomarker findings for TMZ and future candidate drugs for GBM treatment.

A platform that can predict drug sensitivities of individual tumours may also advance the fulfilment of patient-tailored cancer treatment. Indeed, our data shows that interpatient variability in GBM hallmark gene expression levels is, in general, well-retained in the derived cell cultures. However, genomics-based targeted therapies have thus far not significantly improved outcome of GBM patients [59]. One cause for this failure is the redundancy in compensatory mechanisms that these highly heterogeneous tumours possess, making it difficult to predict efficacy of a targeted agent [60]. Personalized drug screening of individual tumours offers a tool to capture the functional effects of candidate drugs on the heterogeneous tumour population, irrespective of the genomic make-up of the tumour, therewith providing prediction of drug efficacy as well as the opportunity to identify alternative (combination) treatment options. However, prior to implementing stratified cohorts based on GSC screening, an additional prospective cohort study should be performed to validate the findings of this study, and confirmation of the assay’s predictive value by other laboratories would contribute to its validity.

Taken together, despite a contextual shift in growing conditions from the natural tumour microenvironment to in vitro culture conditions, patient-derived GBM 2D ex vivo models offer a valuable tool in preclinical research and functional drug screening may aid in identifying effective therapies for subgroups or individual patients. With the implementation of such model systems in drug development programs, it is expected that promising agents will also perform better in (stratified) clinical trials, therewith improving the outlook for GBM patients.

### Supplementary information

Supplemental Figures

Supplemental Tables 1–6

Supplemental file 1

Supplemental legends for Figures and Tables

Supplemental Methods

## Data Availability

RNA sequencing data of the cell lines (*n* = 19) applied in this article is available in the Gene Expression Omnibus Repository: https://www.ncbi.nlm.nih.gov/geo/query/acc.cgi?acc=GSE232173, Accession number: GSE232173. RNA sequencing data of associated parental tissues of Erasmus MC data set (*n* = 56) are available from the corresponding author upon reasonable request. RNA sequencing data of primary tissue data of the complete GLIOTRAIN data set (*n* = 126) will be made available in the European Genome-Phenome Archive Repository, pending completion of statutory trans-national confidentiality checks. The R code used to generate the results in this publication are freely available from our github: https://github.com/ErasmusMC-Bioinformatics/tmz-analysis under the GNU General Public License v.3.0.
