# Supplementary material for: Ex vivo drug sensitivity screening predicts response to temozolomide in glioblastoma patients and identifies candidate biomarkers
Source: Br J Cancer. 2023 Aug 24;129(8):1327–38. doi: 10.1038/s41416-023-02402-y (PMC10575865; doi:10.1038/s41416-023-02402-y)

*Supplemental Figure 1:*


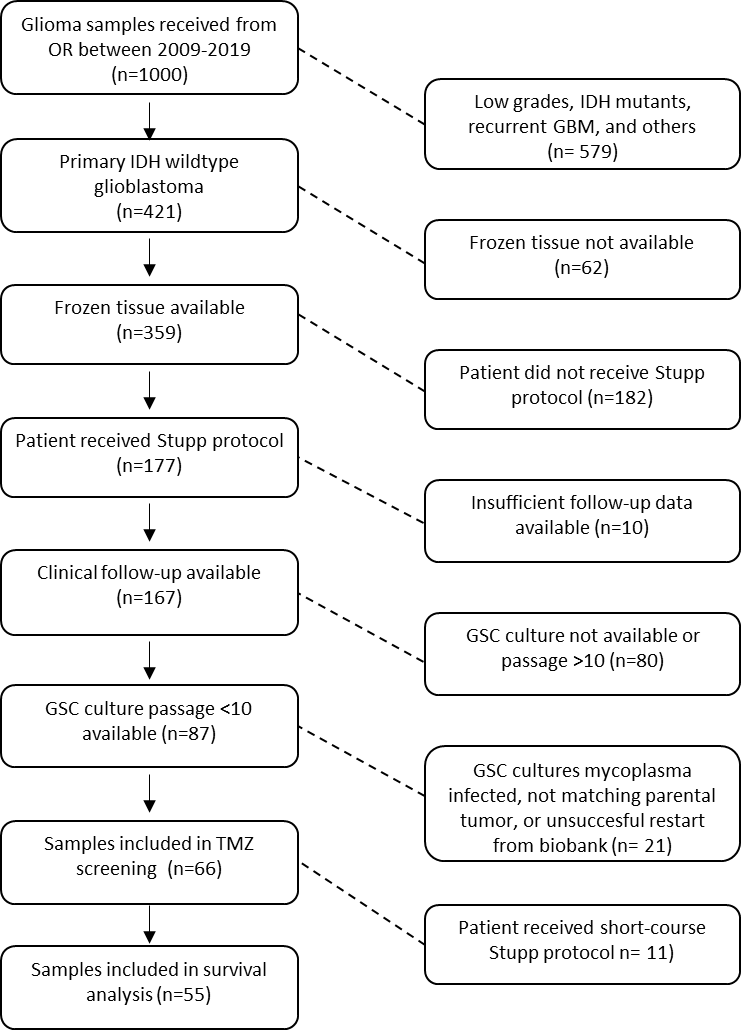


*Supplemental figure 2:*


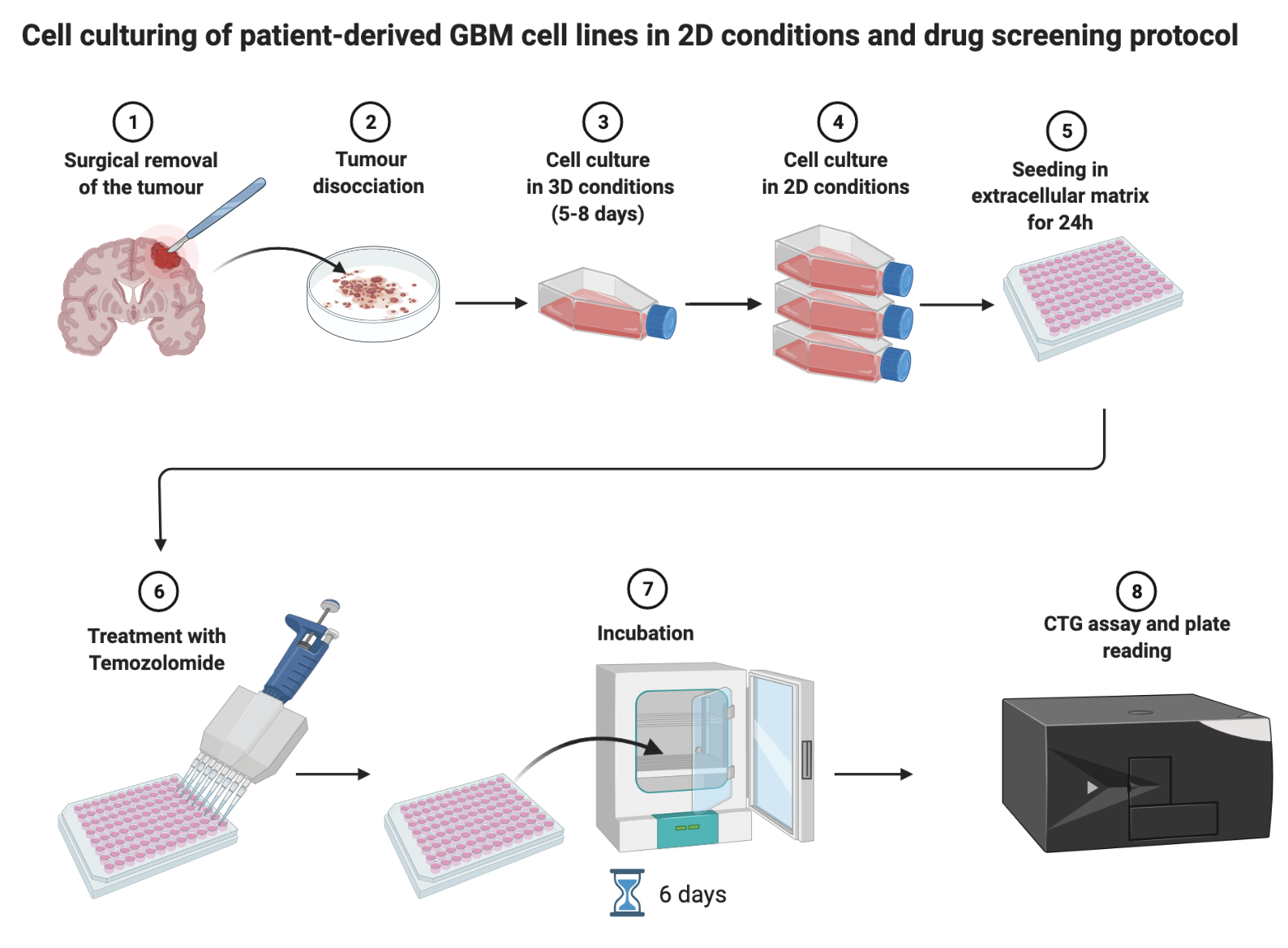


*Supplemental figure 3:*


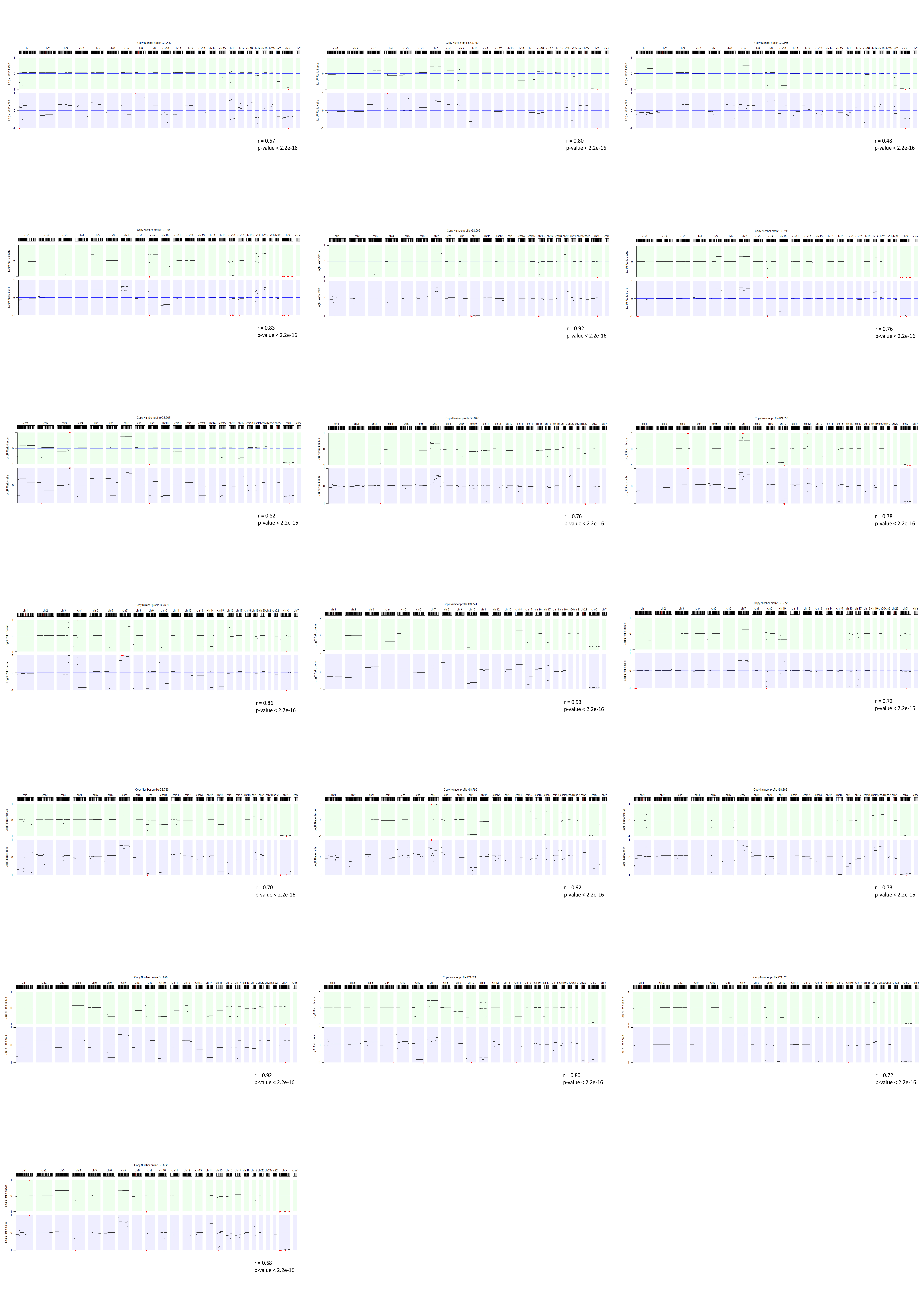


*Supplemental figure 4:*


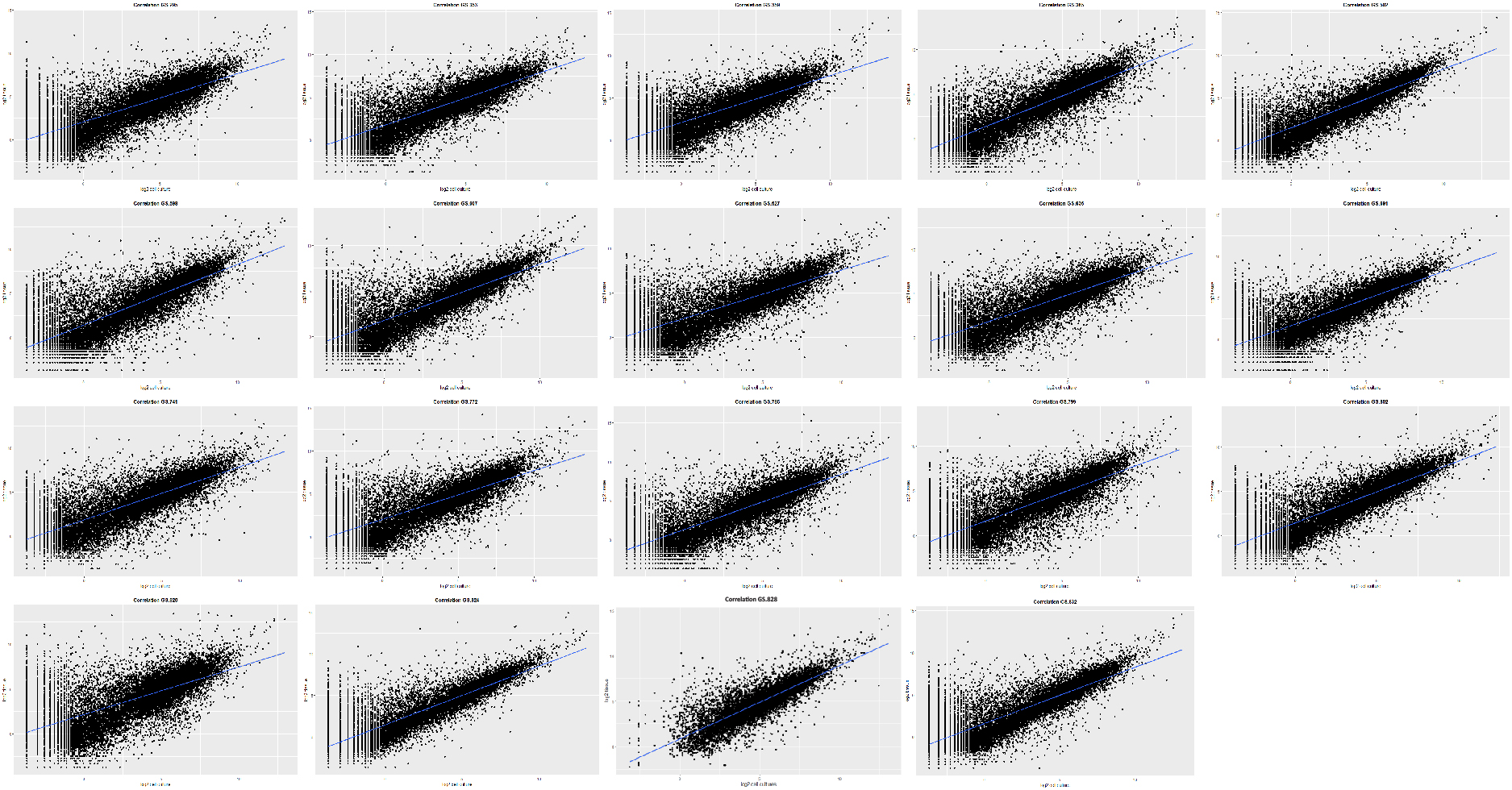


*Supplemental figure 5:*

*
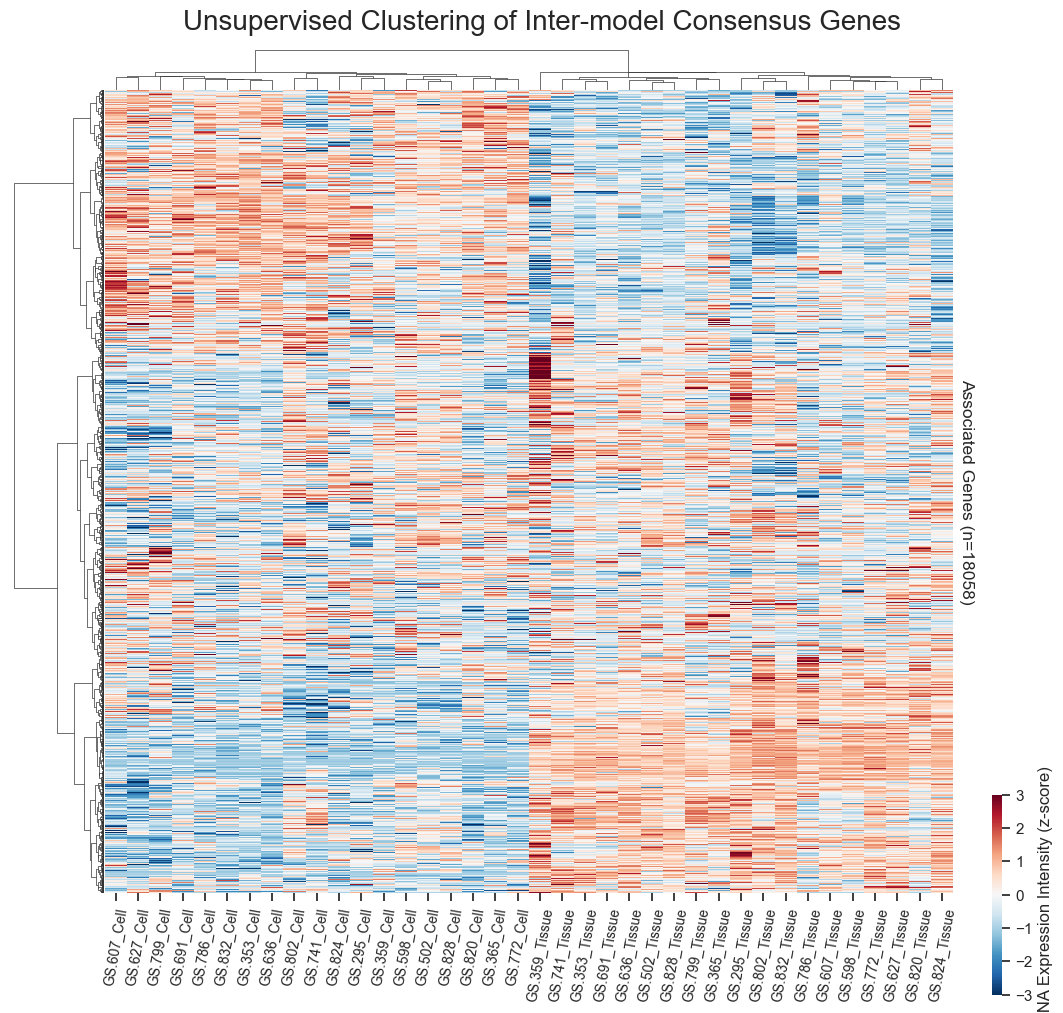
*

*Supplemental figure 6:*

*A*

*
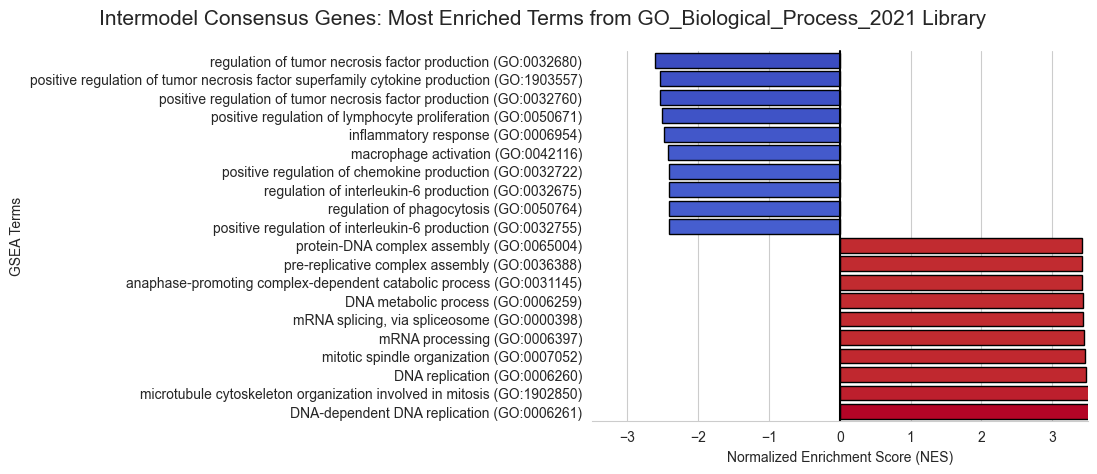
*

*B*

*
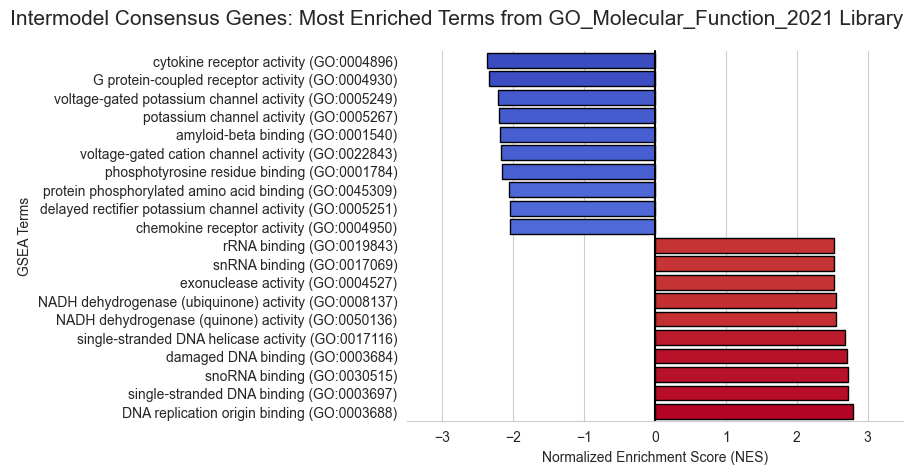
*

*C*

*
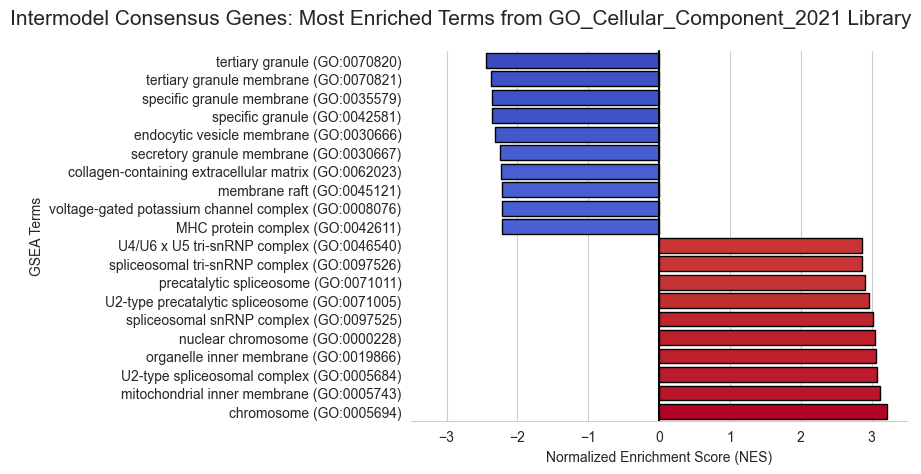
*

*D*

*
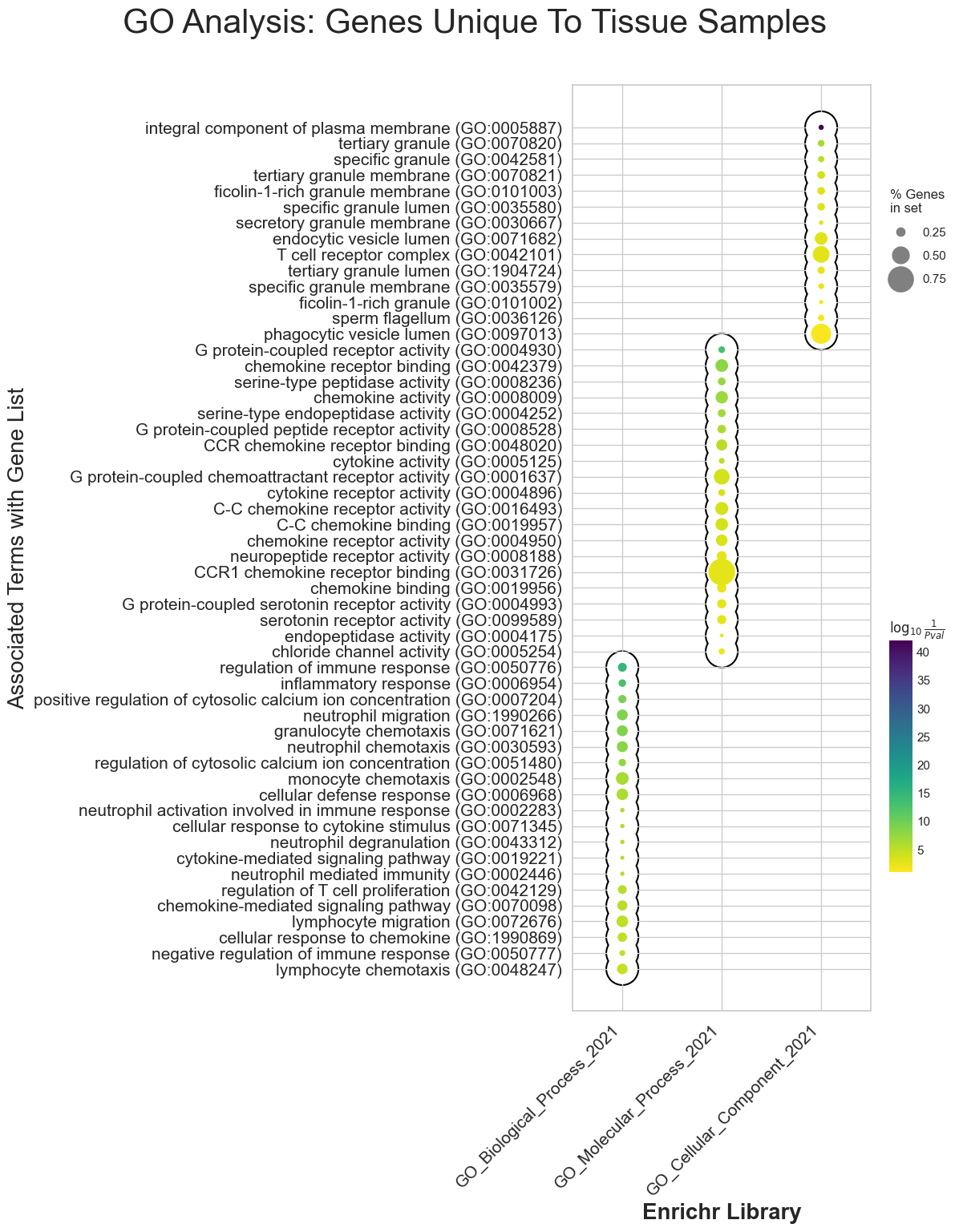
*

*Supplemental figure 7:*


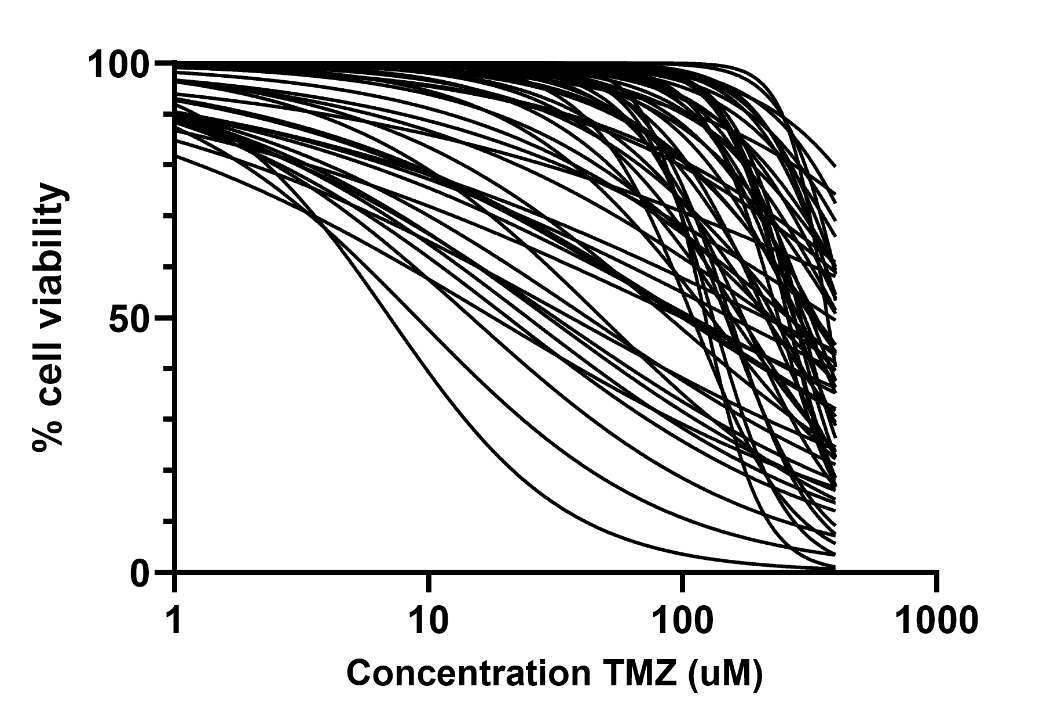


*Supplemental figure 8:*


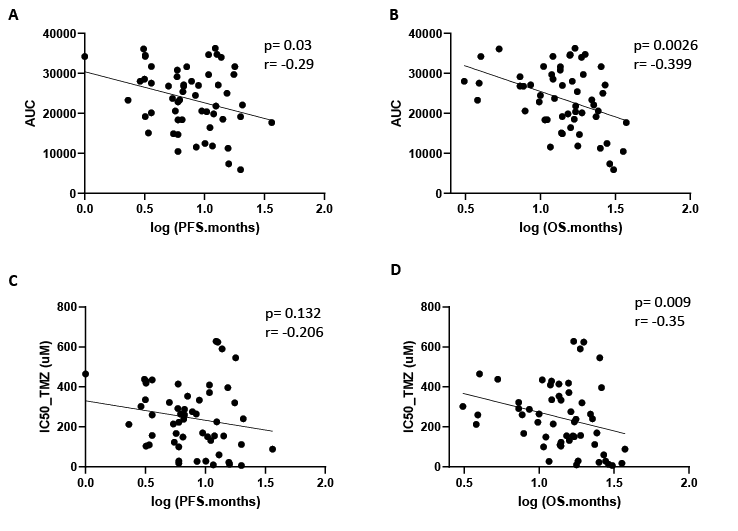


*Supplemental figure 9:*


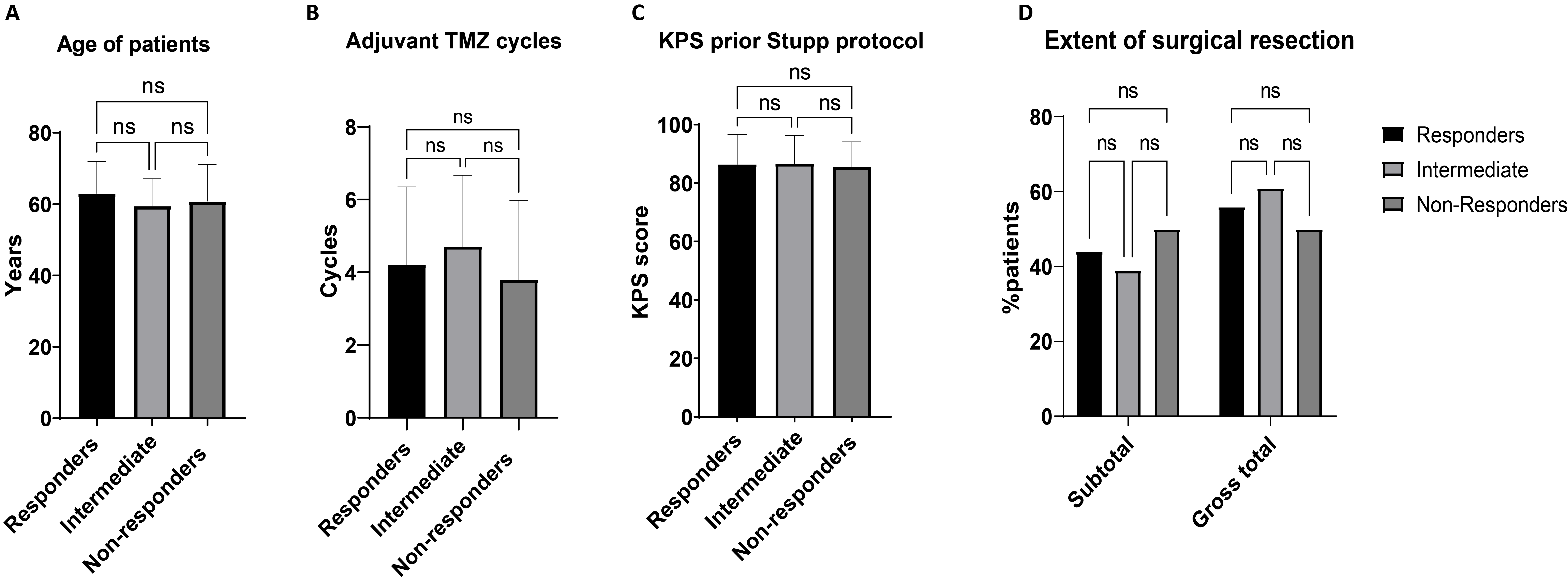


*Supplemental figure 10:*


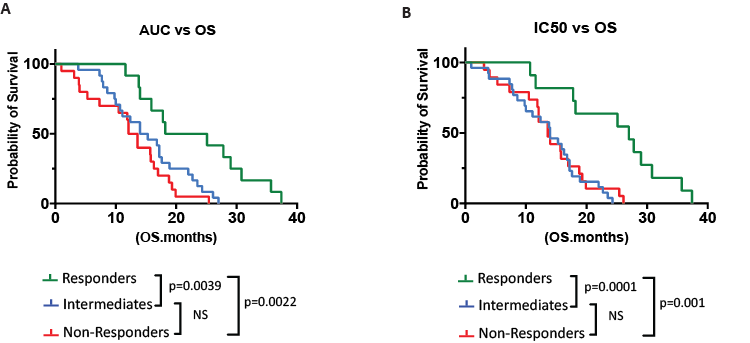


*Supplemental figure 11:*


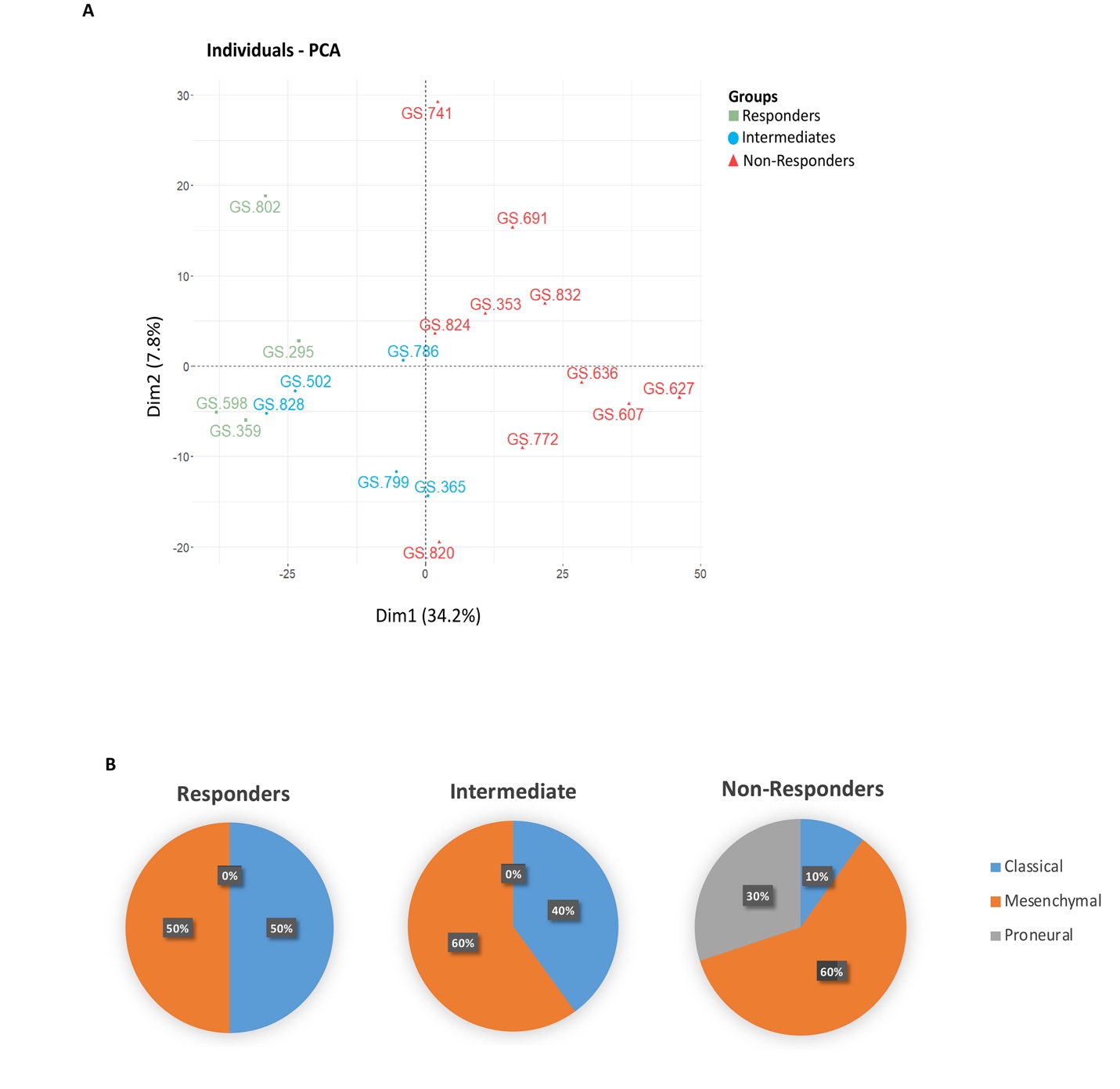


*Supplemental figure 12:*


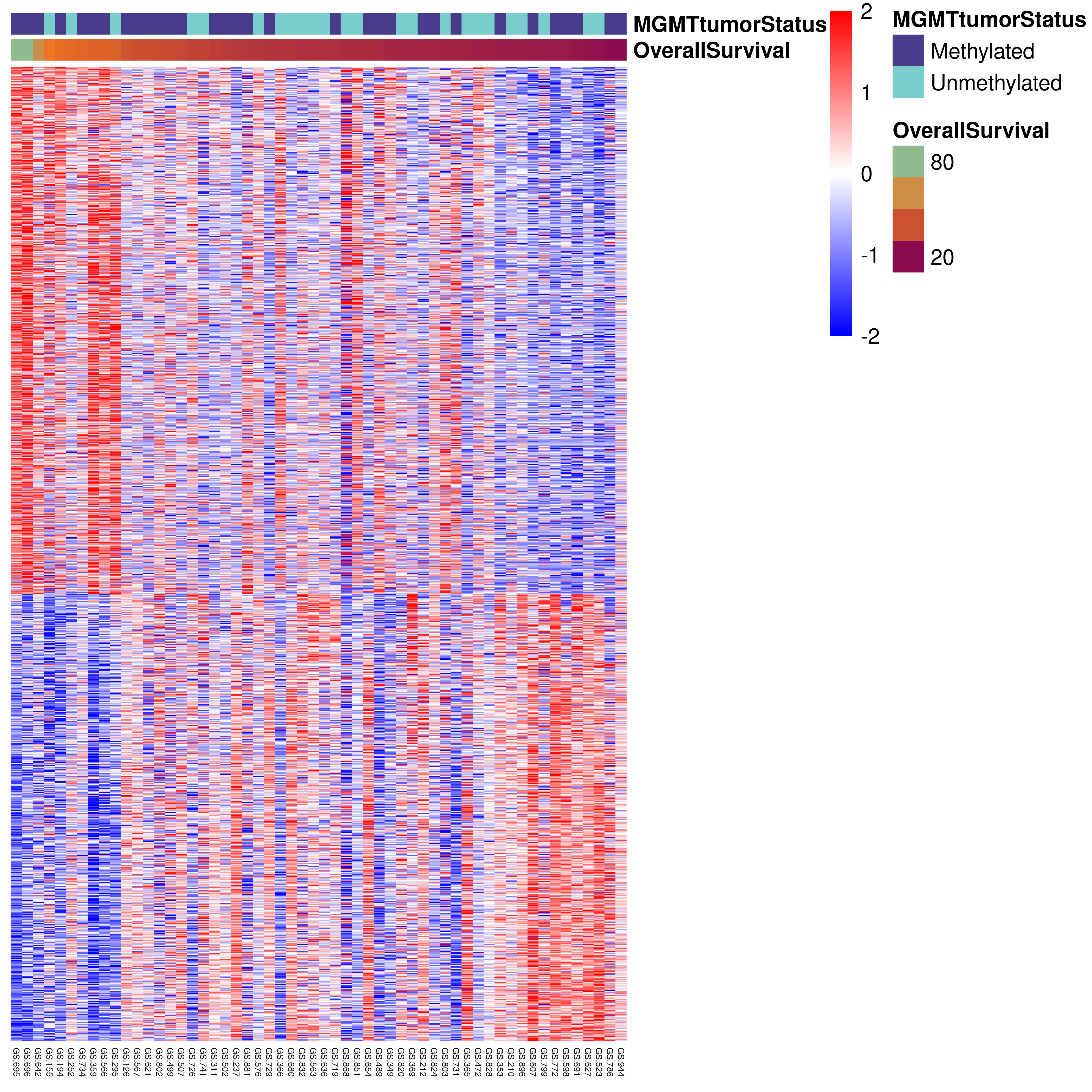

Supplement: Supplementary file 1 — Supplemental Figures [file 41416_2023_2402_MOESM1_ESM.docx]
