# Supplementary material for: Ex vivo drug sensitivity screening predicts response to temozolomide in glioblastoma patients and identifies candidate biomarkers
Source: Br J Cancer. 2023 Aug 24;129(8):1327–38. doi: 10.1038/s41416-023-02402-y (PMC10575865; doi:10.1038/s41416-023-02402-y)
Supplement: Supplementary file 2 — Supplemental Tables 1–6 [file 41416_2023_2402_MOESM2_ESM.docx]

Supplemental table 1: Patient characteristics

| Characteristics | Patients (n=66) |
| --- | --- |
| Age (years) |  |
| median | 61.54 |
| range | 35.4 – 81.03 |
| Sex |  |
| female | 15 |
| male | 51 |
| Surgery |  |
| gross total | 35 |
| subtotal | 30 |
| open biopsy | 1 |
| KPS |  |
| >70 | 55 |
| <70 | 11 |
| Adjuvant cycles of TMZ (n=55) |  |
| median | 5 |
| range | 0 - 8 |
| Short RT/TMZ scheme (n=11) |  |
| median | 0 |
| range | 0-3 |
| MGMT status |  |
| methylated | 31 |
| unmethylated | 35 |

Supplemental table 2: Operators comparison

| GS.number | Operator 1 (IN) | Operator 2 (AA) | Operator 3 (SZ) | Mean %viab 100uM | SD | %CV |
| --- | --- | --- | --- | --- | --- | --- |
| GS184 | 17.7 | 14.6 |  | 16.16 | 2.14 | 13.25 |
| GS274 | 53.7 | 70.9 |  | 62.30 | 12.20 | 19.59 |
| GS281 | 94.3 | 106.8 |  | 100.57 | 8.83 | 8.78 |
| GS295 | 31.0 | 26.6 | 12 | 23.20 | 9.95 | 42.87 |
| GS304 | 55.7 | 67.7 |  | 61.68 | 8.50 | 13.78 |
| GS353 | 75.7 | 72.9 | 75 | 74.54 | 1.42 | 1.91 |
| GS357 | 62.0 | 64.0 |  | 62.98 | 1.39 | 2.21 |
| GS359 | 38.0 |  | 48 | 43.00 | 7.07 | 16.44 |
| GS365 | 74.3 | 81.0 | 99 | 84.78 | 12.76 | 15.05 |
| GS370 | 116.0 | 110.2 |  | 113.11 | 4.09 | 3.61 |
| GS436 | 78.3 | 89.4 |  | 83.87 | 7.84 | 9.35 |
| GS461 | 87.0 | 99 |  | 92.99 | 8.50 | 9.14 |
| GS502 | 69.0 | 72.1 |  | 70.56 | 2.21 | 3.13 |
| GS507 | 72.4 | 41 |  | 56.72 | 22.22 | 39.19 |
| GS523 | 104.0 | 107.9 |  | 105.97 | 2.79 | 2.63 |
| GS528 | 101.3 | 100.8 |  | 101.04 | 0.40 | 0.40 |
| GS597 | 105.0 | 156.8 |  | 130.92 | 36.66 | 28.00 |
| GS607 | 86.7 | 91.5 |  | 89.07 | 3.39 | 3.81 |
| GS616 | 81.7 | 82.0 |  | 81.86 | 0.26 | 0.32 |
| GS622 | 68.0 | 44 |  | 56.00 | 16.97 | 30.30 |
| GS627 | 111.3 | 107.9 | 81 | 100.09 | 16.62 | 16.61 |
| GS630 | 78.3 | 87.6 |  | 82.95 | 6.53 | 7.87 |
| GS636 | 91.9 | 81 |  | 86.47 | 7.74 | 8.95 |
| GS650 | 51.7 | 66.3 |  | 58.96 | 10.31 | 17.49 |
| GS691 |  | 84 | 90 | 87.00 | 4.24 | 4.88 |
| GS741 | 89.0 | 103.9 |  | 96.45 | 10.54 | 10.93 |
| GS772 | 92.3 | 95.1 | 100 | 95.81 | 3.88 | 4.05 |
| GS773 | 33.3 | 57.3 |  | 45.32 | 16.95 | 37.41 |
| GS786 |  | 58.4 | 70 | 64.21 | 8.20 | 12.76 |
| GS820 | 83.3 |  | 76 | 79.63 | 5.13 | 6.44 |
| GS823 | 82.7 | 79.8 |  | 81.24 | 2.02 | 2.49 |
| GS824 | 88.9 |  | 62 | 75.45 | 19.02 | 25.21 |
| GS832 | 92.7 |  | 83 | 87.83 | 6.82 | 7.77 |
| GS851 | 108.3 | 100 |  | 104.17 | 5.89 | 5.65 |
| GS852 | 97.3 | 101 |  | 99.17 | 2.60 | 2.62 |
| GS868 | 72.0 | 74.1 |  | 73.03 | 1.46 | 2.00 |
| GS880 | 50.7 | 47.9 |  | 49.28 | 1.96 | 3.99 |
| GS881 | 91.5 | 108 |  | 99.73 | 11.70 | 11.73 |
| GS883 | 53.0 | 57 |  | 55.00 | 2.83 | 5.14 |
| GS886 | 100.3 | 98.1 |  | 99.22 | 1.57 | 1.58 |

Supplemental table 3: Comparison of tumour and derived cultures

| **A) Correlation DNA sequences** |  |
| --- | --- |

| **GS#** | **r values** | **p-adjusted** |
| --- | --- | --- |
| GS.598 | 0.76 | <2.2e-16 |
| GS.607 | 0.82 | <2.2e-16 |
| GS.627 | 0.76 | <2.2e-16 |
| GS.691 | 0.86 | <2.2e-16 |
| GS.772 | 0.72 | <2.2e-16 |
| GS.786 | 0.70 | <2.2e-16 |
| GS.799 | 0.92 | <2.2e-16 |
| GS.353 | 0.80 | <2.2e-16 |
| GS.365 | 0.83 | <2.2e-16 |
| GS.502 | 0.92 | <2.2e-16 |
| GS.636 | 0.78 | <2.2e-16 |
| GS.741 | 0.93 | <2.2e-16 |
| GS.802 | 0.73 | <2.2e-16 |
| GS.820 | 0.92 | <2.2e-16 |
| GS.824 | 0.80 | <2.2e-16 |
| GS.828 | 0.72 | <2.2e-16 |
| GS.832 | 0.68 | <2.2e-16 |
| GS.295 | 0.67 | <2.2e-16 |
| GS.359 | 0.48 | <2.2e-16 |

| mean | 0.77 |
| --- | --- |
| median | 0.77 |

**B) Correlation RNA sequences**

| **GS#** | **r values** | **p-values adjusted** |
| --- | --- | --- |
| GS.598 | 0.796798718 | <0.0001 |
| GS.607 | 0.808626275 | <0.0001 |
| GS.627 | 0.748419142 | <0.0001 |
| GS.691 | 0.819973968 | <0.0001 |
| GS.772 | 0.757033861 | <0.0001 |
| GS.786 | 0.777949368 | <0.0001 |
| GS.799 | 0.752097317 | <0.0001 |
| GS.353 | 0.801210386 | <0.0001 |
| GS.365 | 0.782467628 | <0.0001 |
| GS.502 | 0.842556383 | <0.0001 |
| GS.636 | 0.766531987 | <0.0001 |
| GS.741 | 0.820844019 | <0.0001 |
| GS.802 | 0.635815509 | <0.0001 |
| GS.820 | 0.863942678 | <0.0001 |
| GS.824 | 0.784002753 | <0.0001 |
| GS.828 | 0.833132849 | <0.0001 |
| GS.832 | 0.750288261 | <0.0001 |
| GS.295 | 0.769702033 | <0.0001 |
| GS.359 | 0.679644459 | <0.0001 |

| Mean 0.77 |  |
| --- | --- |
| Median 0.78 |  |
| Supplemental table 4: Significant testing of hallmark genes in tissue compared to derived cell cultures   \| **Gene #** \| **GBM Hallmark Genes** \| **Wilcoxon Signed Rank P Value** \| **Spearman Correlation Coefficient R Value** \| **Spearman Correlation Coefficient P Value** \| \| --- \| --- \| --- \| --- \| --- \| \| 0 \| PTEN \| 0.000008 \| 0.900449 \| 1.493945e-07 \| \| 1 \| CDKN2A \| 0.000790 \| 0.810982 \| 2.527079e-05 \| \| 2 \| AKT1 \| 0.000004 \| 0.800000 \| 3.923856e-05 \| \| 3 \| MGMT \| 0.001411 \| 0.786550 \| 6.494731e-05 \| \| 4 \| NF1 \| 0.104156 \| 0.724561 \| 4.501976e-04 \| \| 5 \| CDK4 \| 0.001171 \| 0.577193 \| 9.665358e-03 \| \| 6 \| CDKN2B \| 0.000790 \| 0.552675 \| 1.412563e-02 \| \| 7 \| MDM2 \| 0.859573 \| 0.500000 \| 2.925804e-02 \| \| 8 \| EGFR \| 0.000011 \| 0.485965 \| 3.489835e-02 \| \| 9 \| NF2 \| 0.000965 \| 0.447368 \| 5.478933e-02 \| \| 10 \| HIF1A \| 0.373596 \| 0.443860 \| 5.695170e-02 \| \| 11 \| PIK3CA \| 0.650749 \| 0.433333 \| 6.382621e-02 \| \| 12 \| PTCH1 \| 0.768005 \| 0.398246 \| 9.126656e-02 \| \| 13 \| DAXX \| 0.000004 \| 0.392982 \| 9.602691e-02 \| \| 14 \| BRAF \| 0.225323 \| 0.359649 \| 1.304368e-01 \| \| 15 \| ALK \| 0.000523 \| 0.357895 \| 1.324603e-01 \| \| 16 \| IGF1R \| 0.257927 \| 0.347368 \| 1.450661e-01 \| \| 17 \| RB1 \| 0.000420 \| 0.310526 \| 1.956889e-01 \| \| 18 \| TP53 \| 0.009453 \| 0.268421 \| 2.665099e-01 \| \| 19 \| FGFR1 \| 0.040131 \| -0.231579 \| 3.401065e-01 \| \| 20 \| PTCH2 \| 0.000420 \| 0.229825 \| 3.438802e-01 \| \| 21 \| NOTCH1 \| 0.768005 \| 0.219298 \| 3.670279e-01 \| \| 22 \| MET \| 0.275341 \| 0.201754 \| 4.075051e-01 \| \| 23 \| CHEK2 \| 0.000267 \| 0.192982 \| 4.286131e-01 \| \| 24 \| MYC \| 0.012360 \| 0.187719 \| 4.415495e-01 \| \| 25 \| MYCN \| 0.123188 \| 0.182308 \| 4.550587e-01 \| \| 26 \| PDGFRA \| 0.225323 \| 0.170175 \| 4.860995e-01 \| \| 27 \| TRAF7 \| 0.000004 \| 0.152632 \| 5.327517e-01 \| \| 28 \| STAT3 \| 0.768005 \| 0.129825 \| 5.963026e-01 \| \| 29 \| SUFU \| 0.007145 \| 0.114035 \| 6.420436e-01 \| \| 30 \| SETD2 \| 0.008232 \| 0.108772 \| 6.575798e-01 \| \| 31 \| BCL2 \| 0.000004 \| -0.077193 \| 7.534433e-01 \| \| 32 \| ATRX \| 0.465317 \| 0.070175 \| 7.752818e-01 \| \| 33 \| DDX3X \| 0.000011 \| 0.063158 \| 7.972829e-01 \| \| 34 \| PIK3R1 \| 0.000004 \| 0.052632 \| 8.305560e-01 \| \| 35 \| BRCA1 \| 0.000004 \| 0.029825 \| 9.035304e-01 \| \| 36 \| VEGFA \| 0.000038 \| 0.007018 \| 9.772536e-01 \| \| 37 \| CDK6 \| 0.000004 \| 0.000000 \| 1.000000e+00 \| |  |

Supplemental table 5: Survival rates

| Survival rates | Time (months) |
| --- | --- |
| median PFS | 6.65 (1.0 - 36.40) |
| UM | 8.55 |
| M | 5.92 |
| median OS | 13.69 (2.8 - 37.40) |
| UM | 12.40 |
| M | 17.19 |

Supplemental table 6: Statistical outcome of survival analysis

| **Cox regression analysis** |  |  |
| --- | --- | --- |
| **Read-outs** | **p.value (PFS)** | **p.value (OS)** |
| 1. % cell viability @100µM | 0.012 | <0.00005 |
| 2. AUC | 0.092 | 0.001 |
| 3. IC_50_ | NS | 0.0004 |
| MGMT: M vs UM | NS | 0.001 |
| **Log-rank test** |  |  |
| **1. % cell viability at 100uM** | **p.value (PFS)** | **p.value (OS)** |
| responders vs intermediate | NS | 0.0023 |
| responders vs non-responders | 0.018 | 0.0001 |
| intermediate vs non-responders | NS | 0.011 |
| **2. AUC** | **p.value (PFS)** | **p.value (OS)** |
| responders vs intermediate | ND | 0.0039 |
| responders vs non-responders | ND | 0.0022 |
| intermediate vs non-responders | ND | NS |
| **3. IC_50_** | **p.value (PFS)** | **p.value (OS)** |
| responders vs intermediate | ND | 0.0001 |
| responders vs non-responders | ND | 0.001 |
| intermediate vs non-responders | ND | NS |
| **4. MGMT status** |  |  |
| Methylated vs unmethylated | 0.0074 | 0.001 |
