# Supplementary material for: Ex vivo drug sensitivity screening predicts response to temozolomide in glioblastoma patients and identifies candidate biomarkers
Source: Br J Cancer. 2023 Aug 24;129(8):1327–38. doi: 10.1038/s41416-023-02402-y (PMC10575865; doi:10.1038/s41416-023-02402-y)
Supplement: Supplementary file 4 — Supplemental legends for Figures and Tables [file 41416_2023_2402_MOESM4_ESM.docx]

**SUPPLEMENTAL FIGURE LEGENDS**

*Supplemental Figure 1: Flow diagram of sample selection from GSC biobank.*

*Supplemental Figure 2: Workflow of patient-derived GBM cell culturing and drug screening platform.*

*Supplemental figure 3: Copy number variation plots of paired tissue and derived cell cultures (n=19).*

*Supplemental figure 4: Spearman’s correlation of transcriptome of paired tissue and derived cell cultures (n=19).*

*Supplemental figure 5: Unsupervised clustering of consensus genes.*

Heatmap showing the delineation between cell cultures and tissues in a set of 18,058 genes expressed in both parental tumors and cell cultures.

*Supplemental figure 6: Molecular comparison of GBM tissues and derived GSC cultures.*

A-C) GSEA on the consensus genes (n=18,058) using 3 reference libraries (GO series) illustrates the top 20 terms with the most positively (n=10) and negatively (n=10) NES. Blue indicates terms with negative enrichment scores and red positive enrichment scores. Intensity of the colour is indicative of relative significance (all terms present are NOM p-val, FDR q-val, FWER p-val significant). D) GO analysis of genes unique to tissue samples. The dot plot shows the associated terms of genes unique to tissue (y-axis) in reference to three different GO libraries (x-axis). The circumference of the dot indicates percentage of the genes present in the associated term and the colour indicates the level of significance. All p-values >0.05 were considered significant.

*Supplemental figure 7: Dose-response curves of temozolomide (TMZ).*

Dose response curves of temozolomide on primary GBM cultures (n=66) showing percentage viability of controls (y axis) and applied TMZ dose in μM (x axis).

*Supplemental figure 8: In vitro response to TMZ correlates with clinical outcome of patients receiving Stupp protocol.*

A-B) Comparison of AUC values for TMZ response (y-axis) of GSC cultures (n=55) with progression free survival (x-axis, log transformed PFS in months) or overall survival (x-axis, log transformed OS in months) of the corresponding patients. C-D) Comparison of IC_50_ values (y-axis) of GSC cultures (n=55) with progression free survival (x-axis, log transformed PFS in months) or overall survival (x-axis, log transformed OS in months) of the corresponding patients. Significant correlation between in vitro response and PFS or OS is considered when p < 0.05 (Spearman’s rank correlation)

*Supplemental figure 9: Age, Adjuvant TMZ cycles, KPS score of patients and extent of surgical resection categorized in the response groups.*

A) Age of patients in the cohort, B) Number of adjuvant TMZ cycles and C) KPS scores of the patients and D) Extent of surgical resection in the three response groups (responders =10, intermediates =17, non-responders =28).

*Supplemental figure 10: In vitro response of TMZ predicts clinical response of GBM patients.*

A-B) Overall survival graphs of the patient cohort (n=55) divided into 3 response categories, responders (green), intermediates (blue) and non-responders (red), based on in vitro sensitivity to TMZ as expressed by AUC or IC50. P values of log-rank analyses comparing the different overall survival curves are noted in the graph (x axis, OS in months). P values < 0.05 were considered significant.

*Supplemental figure 11: Distribution of the GBM transcriptomic subtypes within TMZ response groups.*

Pie-charts showing the percentages of GBM transcriptomic subtypes classical (blue), mesenchymal (orange) and proneural (grey) within the three *in vitro* TMZ response groups (responders, intermediate and non-responders).

*Supplemental figure 12: Transcriptome correlations of patient response to TMZ/RTx treatment.*

Heatmap of the top 100 signature genes correlated with overall survival (in months) of 56 patients treated with TMZ/RTx (*p*-value < 0.05). Each row of the heatmap represents one significant gene, while each column represents one sample. The genes are ordered by the HR values of the CoxPH analysis. The samples are ordered by increasing overall survival time.

**SUPPLEMENTAL TABLE LEGENDS**

*Supplemental table 1: Patient characteristics*

Patient characteristics of samples included in study (n=66)

*Supplemental table 2: Operators comparison*

Table showing temozolomide sensitivity data (n=40 cell cultures) generated by two or three different operators and the calculated percentage of coefficient of variation (CV%).

*Supplemental table 3: Comparison of tumor and derived cultures*

Tables showing correlation coefficients for RNA (n=19) and DNA (n=19) sequences of GSC lines compared to parental tumor tissues

*Supplemental table 4: Significance testing of hallmark genes in tissue compared to derived cell cultures.*

Table showing outcome of Wilcoxon Signed Rank and Spearman correlation tests on the expression of hallmark genes (n=37) between paired parental tissue and derived cell cultures (n=19).

*Supplemental table 5: Survival rates*

Median PFS and OS in relation to MGMT methylation status for our cohort (n=66)

*Supplemental table 6: Statistical outcome of survival analysis*

Tables showing log-rank and Cox regression analysis outcome for PFS and OS, by gradient and by response classification.
