# Supplementary material for: Ex vivo drug sensitivity screening predicts response to temozolomide in glioblastoma patients and identifies candidate biomarkers
Source: Br J Cancer. 2023 Aug 24;129(8):1327–38. doi: 10.1038/s41416-023-02402-y (PMC10575865; doi:10.1038/s41416-023-02402-y)
Supplement: Supplementary file 5 — Supplemental Methods [file 41416_2023_2402_MOESM5_ESM.docx]

# Supplementary methods:

## Tumor processing and cell culture

Resected tumor samples are collected from the operation room in plain culture medium (DMEM, Gibco, Thermo Fisher Scientific) and kept at 4°C for a maximum of 4 hours until processing. Tissue is first mechanically dissociated with a scalpel removing necrotic areas and large vasculature. Tumor fragments are then enzymatically dissociated with Collagenase A and DNAse (both from Roche). Red blood cells are removed by incubating the pellets with an erythrocyte lysis buffer. Single cells and small tissue fragments are then resuspended in culture medium (DMEM-F12), supplemented with penicillin/streptomycin, B27, 20 ng/mL bFGF, 20 ng/mL EGF (all from Gibco, Thermo Fisher Scientific), 5 μg/mL Heparin (Alfa Aesar) and transferred to uncoated culture flasks. All cultures are kept at 37°C in a humidified incubator with 5% CO_2_. During 5-8 days the formation of neurospheres takes place which are then transferred to a new flask coated with 1:100 diluted Cultrex PathClear Reduced Growth Factor BME (R&D Systems). Neurospheres attach and transition to monolayers during 1-2 weeks. Cultures are split when >90% confluency is reached or if there are multiple areas in the flasks of very high cell density.

Cultures were regularly tested for mycoplasma infection using the MycoAlertTM PLUS Mycoplasma Detection Kit (Lonza) and consistency between tumor and derived cell cultures was verified by short tandem repeat-based genotyping using Geneprint (Promega).

## In vitro temozolomide testing

Cells were plated at 1000 cells/well in triplo into extracellular matrix coated (BD Bioscience) 96-well plates and incubated for 24 hours. Temozolomide was dissolved in DMSO to make a stock solution of 0.1M. The stock solution was diluted in serum-free culture medium to obtain a starting concentration of 400uM which was further diluted in 2-fold steps until reaching the lowest tested concentration of 6.25uM. The highest DMSO concentration was 0.4% and the same 2-fold dilution steps were applied for the controls. Serial dilutions of TMZ and DMSO in medium were applied as controls. Six days after treatment cell viability was determined using the ATP-based Cell Titer Glo Assay (Promega) according to manufacturer’s instructions. This assay was previously found to provide the most specific and reproducible readout for drug screening on primary GSCs [1]. Luciferase signals were measured with the Tecan Infinite 200 (Tecan). Viability was calculated as a percentage of matched DMSO controls and 2 or 3 biological replicates were performed.

## RNA sequencing

Tissue was removed from Allprotect solution (Qiagen) and placed in a 1.5mL microcentrifuge tube. 0.5mL of Trizol (Thermo Fisher scientific) was added to each sample and incubated for >10 minutes, before total RNA extraction via addition of 100 uL of chloroform. The solution was vortexed for 10 seconds and centrifuged at 12,000 x g for 10 minutes at 4^o^C. The supernatant was transferred to a fresh microfuge tube and 250uL of isopropanol added. and incubated for 3 minutes. This was centrifuged at 12,000 x g for 10 minutes at 4^o^C. The supernatant was discarded, the pellet washed twice in 1 mL 75% ethanol. The supernatant was discarded and pellet was allowed to dry. The pellet was dissolved in 40 ul of RNAase free water. RNA was quantified using using the NanoDrop UV-Vis spectrophotometer (Thermo Fischer Scientific).

Transcriptome analyses of tissues versus derived cell cultures

For this analysis, the transcriptome of 19 tissues from Erasmus GLIOTRAIN cohort and their derived cell cultures (n=19) were applied. Inter-model consensus genes (n = 18,058) were isolated by identifying genes only expressed in both the normalized tissue and derived cell culture datasets, utilizing matching samples that were preprocessed and normalized as previously described. Pandas [2], Seaborn [3] and SciPy [4] packages were utilized for unsupervised clustering of all inter-consensus genes utilizing Euclidean distancing and Ward's method. Within the clustermap, per gene expression was standardized across datasets utilizing z-score.

Gene Set Enrichment Analysis (GSEA) was performed with the use of GSEApy [5]. For GSEA, the previously mentioned normalized tissue and cell culture datasets were utilized. GSEA was performed on the inter-model consensus genes utilizing the GO_Biological_Process_2021, GO_Molecular_Function_2021, GO_Cellular_Component_2021, and MSigDB_Hallmark_2020 libraries hosted on Enrichr [6-11]. Significant terms were isolated through the combined use of significant (p ≤ 0.05) nominal (Nom) p-values, false discovery rate (FDR) q-values, and family-wise error rate (FWER) p-values. Terms with the most extreme NES scores (10 positive, 10 negative) were visualized utilizing Seaborn’s barplot [3]. On a per library basis, a gene list was generated from all significant terms, and utilized for unsupervised clustering as previously described. Genes which were associated with multiple terms were designated with the term label “Multiple Associated Terms”. The top 4 most positively NES and negatively NES terms were used to generate a summary GSEA clustermap highlighting transcriptomic differences between tissue and cell cultures with matching parental material.

Over-representation analysis was completed via GSEApy [12] on a gene list containing genes unique to the tissue model samples and not found within the cell culture samples. The GO_Biological_Process_2021, GO_Molecular_Function_2021, and GO_Cellular_Component_2021 libraries to provide insight into the transcriptomic composition of genes unique to tissue [13]. Results were visualized using GSEApy’s built-in dotplot functionality.

Comparative analysis of GBM hallmark genes’ expression between cell culture and tissue datasets, were performed with a custom script utilizing Seaborn [3], Matplotlib [13], NumPy [12], and SciPy [4]. Significance of differences within expression were evaluated via Wilcoxon Signed Rank and Spearman's Correlation Coefficient tests functions within SciPy. Genes designated as hallmark for IDHwt GBM are those utilized within Erasmus MC pathology department for tumor classification.

Correlation analysis of the TMZ response with RNA-seq data of cell cultures and tumor tissues

Differential expression analysis was conducted to compare the expressed genes in cell culture and tissue RNA-seq data. For cell culture data (n=19) we applied the identified response groups based on % viability at 100uM TMZ. For tissue data, samples derived from the Erasmus GLIOTRAIN cohort (n=56) were labeled as 'Responders' if OS> 36 months (n=10), ‘Intermediates’ if OS between 10-36 months (n=36) and as 'Non-Responders' if the overall survival was less than 10 months (n=10). Lowly expressed genes were filtered out by setting count data of ≤ 4 to zero, and removing genes that were expressed in less than 10% of samples. Differentially expressed genes (DE genes) between responding and non-responding groups were identified using the DESeq2 package in R [14]. DESeqDataSet was constructed using the "DESeqDataSetFromMatrix" function. DEG analysis was performed with the "DESeq" function using a p-value threshold of 0.05.

A Spearman correlation analysis was performed with the R function “cor.test”[15] on the significant DE genes of the cell culture to determine which genes are correlated with TMZ response. In this analysis, all cell culture data, including responders, intermediates, and non-responders, with a total sample size of n=19, was used. The raw count matrix was normalized using the TMM method in log2 scale, utilizing the "CalcNormFactors" function from the "EdgeR" package [16].

A Cox proportional hazards (CoxPH) analysis was performed, with the R function “survival” [17], on the significant DE genes of tumour data set to determine which of the identified DE genes have an effect on the survival outcome or TMZ response in patients. All tissue data, including responders, intermediates, and non-responders, with a total sample size of n=56, was used. The raw count data was normalized and log-transformed as described in the correlation analysis.

A Cox proportional hazards (CoxPH) analysis was performed on the overlapping genes of the cell culture and tissue data sets as validation on two additional cohorts: the GLIOTRAIN dataset (excluding samples derived from the Erasmus MC) with a total of n=70 samples, and the TCGA dataset selected for GBM patients treated with TMZ of n=89 samples. The raw count matrices in both datasets were normalized and log-transformed as described in the correlation analysis.

## References

1. Kleijn, A., et al., A Systematic Comparison Identifies an ATP-Based Viability Assay as Most Suitable Read-Out for Drug Screening in Glioma Stem-Like Cells. Stem cells international, 2016. **2016**: p. 5623235-5623235.
2. McKinney, W., & others. (2010). Data structures for statistical computing in python. In Proceedings of the 9th Python in Science Conference (Vol. 445, pp. 51–56).
3. Waskom, M. L., (2021). seaborn: statistical data visualization. Journal of Open Source Software, 6(60), 3021, https://doi.org/10.21105/joss.03021
4. Pauli Virtanen, Ralf Gommers, Travis E. Oliphant, Matt Haberland, Tyler Reddy, David Cournapeau, Evgeni Burovski, Pearu Peterson, Warren Weckesser, Jonathan Bright, Stéfan J. van der Walt, Matthew Brett, Joshua Wilson, K. Jarrod Millman, Nikolay Mayorov, Andrew R. J. Nelson, Eric Jones, Robert Kern, Eric Larson, CJ Carey, İlhan Polat, Yu Feng, Eric W. Moore, Jake VanderPlas, Denis Laxalde, Josef Perktold, Robert Cimrman, Ian Henriksen, E.A. Quintero, Charles R Harris, Anne M. Archibald, Antônio H. Ribeiro, Fabian Pedregosa, Paul van Mulbregt, and SciPy 1.0 Contributors. (2020) SciPy 1.0: Fundamental Algorithms for Scientific Computing in Python. Nature Methods, 17(3), 261-272.
5. Zhuoqing Fang, Xinyuan Liu, Gary Peltz, GSEApy: a comprehensive package for performing gene set enrichment analysis in Python, Bioinformatics, 2022;, btac757, <https://doi.org/10.1093/bioinformatics/btac757>
6. Ashburner et al. Gene ontology: tool for the unification of biology. Nat Genet. May 2000;25(1):25-9.
7. The Gene Ontology resource: enriching a GOld mine. Nucleic Acids Res. Jan 2021;49(D1):D325-D334.
8. Liberzon A, Birger C, Thorvaldsdóttir H, Ghandi M, Mesirov JP, Tamayo P. The Molecular Signatures Database (MSigDB) hallmark gene set collection. Cell Syst. 2015 Dec 23;1(6):417-425. doi: 10.1016/j.cels.2015.12.004. PMID: 26771021; PMCID: PMC4707969.
9. Chen EY, Tan CM, Kou Y, Duan Q, Wang Z, Meirelles GV, Clark NR, Ma'ayan A.

Enrichr: interactive and collaborative HTML5 gene list enrichment analysis tool. BMC Bioinformatics. 2013; 128(14).

1. Kuleshov MV, Jones MR, Rouillard AD, Fernandez NF, Duan Q, Wang Z, Koplev S, Jenkins SL, Jagodnik KM, Lachmann A, McDermott MG, Monteiro CD, Gundersen GW, Ma'ayan A.Enrichr: a comprehensive gene set enrichment analysis web server 2016 update. Nucleic Acids Research. 2016; gkw377.
2. Xie Z, Bailey A, Kuleshov MV, Clarke DJB., Evangelista JE, Jenkins SL, Lachmann A, Wojciechowicz ML, Kropiwnicki E, Jagodnik KM, Jeon M, & Ma’ayan A.

Gene set knowledge discovery with Enrichr. Current Protocols, 1, e90. 2021. doi: 10.1002/cpz1.90

1. Harris, C.R., Millman, K.J., van der Walt, S.J. et al. Array programming with NumPy. Nature 585, 357–362 (2020). DOI: 10.1038/s41586-020-2649-2
2. J. D. Hunter, "Matplotlib: A 2D Graphics Environment", Computing in Science & Engineering, vol. 9, no. 3, pp. 90-95, 2007.
3. Love MI, Huber W, Anders S (2014). “Moderated estimation of fold change and dispersion for RNA-seq data with DESeq2.” Genome Biology, 15, 550. doi:10.1186/s13059-014-0550-8.
4. Dodge Y. Spearman Rank Correlation Coefficient, in The Concise Encyclopedia of Statistics. 2008, Springer New York: New York, NY. p. 502-505
5. Robinson MD, McCarthy DJ, Smyth GK (2010). “edgeR: a Bioconductor package for differential expression analysis of digital gene expression data.” Bioinformatics, 26(1), 139-140. doi:10.1093/bioinformatics/btp616.
6. Therneau, T.M., A Package for Survival Analysis in R. 2022.
